# Proceedings of the 4th Annual PROMIS® Health Organization Conference: Global Advances in Methodology and Clinical Science: Dublin, Ireland. 28-29 October 2018

**DOI:** 10.1186/s41687-018-0079-9

**Published:** 2018-11-30

## O001 Using PROMIS-29 to Examine How Caring for a Child with a Health Condition Affects Health of Caregivers

### Dagmar Amtmann ^1^, Mara Nery-Hurwit^1^, Alyssa Bamer^1^, Rana Salem ^1^, Arnold R. Gammaitoni^2^, Carey R. Aron^2^, Bradley S. Galer^2^, Mark P. Jensen^1^

#### ^1^Department of Rehabilitation Medicine, University of Washington, Seattle, WA, USA; ^2^Zogenix, Medical Affairs, 5858 Horton Street, Suite 455, Emeryville, CA 94608, USA

**Objective**

Caregiving for children affects caregivers’ lives in many significant ways. Stress of caregivers is understudied and under-reported. The objective was to examine how taking care of a child (<18 years old) affects health of caregivers with mostly typically developing children versus children with a medical condition as a first step toward developing a screener to identify caregivers who need additional supports.

**Methods**

Caregivers responded to PROMIS-29 and the University of Washington Caregiver Stress Scale (UW-CSS), a self-reported IRT-based item bank. For all scales, the general population score is 50. The samples included a community sample, and caregivers of children with Epileptic Encephalopathies (i.e., severe epilepsy), Muscular Dystrophy (MD) and Down Syndrome (DS). The clinical populations were selected because they represent different types of caregiving stress (e.g., mostly cognitive or physical challenges, or both). The mean scores were compared between the general population and clinical populations using t-tests.

**Results**

Data from a total of 722 caregivers were used; community sample (n=322), DS (n=143), MD (n=129), and epilepsy (n=128). Average age of caregivers was 42 years (SD=9), 83% were female, 82% were white, 73% were married, 17% had high school education or less, 41% were employed full time, and 90% were biological parents. The average child age was 9 years (SD=5). The same pattern emerged across different health domains, including caregiver stress, with the community sample caregivers reporting better health and less stress than any of the clinical samples. The worst caregiver stress was reported by caregivers of children with epilepsy (M=63). Compared to the community sample, PROMIS scores were substantially worse (> 5 points) for anxiety and fatigue (epilepsy, DS, MD), sleep (epilepsy), social (epilepsy) and depression (epilepsy and MD).

**Conclusions**

Compared to the community sample, caregivers of children with medical conditions report considerably worse health.

**Funding**

This investigator-initiated study was supported by Zogenix and in part by a grant from the National Institute on Disability, Independent Living, and Rehabilitation Research H133P120002.

**Keywords**

Caregiver Stress, IRT, QOL, PRO, PROMIS 29, Epilepsy, Down Syndrome, Muscular Dystrophy

## P002 Evaluating the Construct Validity of PROMIS Fatigue Short Forms in Rheumatoid Arthritis

### Susan J. Bartlett^1,2^, A.^.^Kristina Gutierrez^2^, Alessandra Butanis^2^, Vivian P. Bykerk^3^, Jeffrey R. Curtis^4^, Amye Leong^5^, Anne Lyddiatt^6^, Ana Maria Orbai^2^, Michelle Jones^2^, W. Benjamin Nowell, and Clifton O. Bingham^2^

#### ^1^McGill University, USA; ^2^Johns Hopkins Medicine, USA; ^3^Hospital for Special Surgery, USA; ^4^University of Alabama at Birmingham, USA; ^5^Healthy Motivation, USA; ^6^Patient Partners, USA

**Background**

Fatigue is prevalent, severe and one of the most disabling symptoms in rheumatoid arthritis (RA). There is no standardized measure for its assessment nor data concerning the performance of PROMIS-Fatigue short forms (SFs) in people with RA. We evaluated the construct validity of 4-, 7-, and 8-item PROMIS-Fatigue SFs in RA patients across the range of disease activity.

**Methods**

Adult RA patients were recruited from an online arthritis patient community and an observational cohort drawing from three academic medical centers. Measures included PROMIS-Fatigue SFs (7a, 8a, 4a), other PROMIS measures of RA symptoms, and selected patient reported outcomes including RAND-36 Vitality, Fatigue NRS, and patient global assessment of disease activity. Clinical outcomes from the observational cohort included swollen and tender joint counts (28), physician global assessment, and the RA Clinical Disease Activity Index (CDAI).

**Results**

A total of 548 (200 online, 348 observational cohort) participants were included. PROMIS Fatigue SF scores spanned the measurement continuum and correlated highly with each other (r’s ≥0.91) and other fatigue measures (r’s ≥0.85). PROMIS-Fatigue SF scores were highly and inversely associated with Physical Function and Participation (r’s -0.77 to -0.78), and moderately-highly and positively correlated with pain, sleep disturbance, anxiety, and depression (r’s 0.60 to 0.75). PROMIS-Fatigue SF scores showed dose-response relationships across fatigue severity descriptors and CDAI categories.

**Conclusions**

These results provide new evidence supporting the construct validity of the 4, 7, and 8-item PROMIS-Fatigue SFs. The SFs capture fatigue across the spectrum of RA disease activity in diverse groups of individuals and should be considered for use as patient-centered assessments of RA disease control and treatment efficacy.

**Funding**

PCORI IP2-PI0000737 and SC14-1402-10818 and NIH P30-AR053503 and AR070254. All statements and conclusions are those of the authors and do not necessarily reflect those of PCORI, its Board of Governors, or Methodology Committee.

**Keywords**

Fatigue, Validity, PRO, Rheumatoid Arthritis, Outcomes

## O003 Combining Online and In-Person Evaluations of Content Relevance of PROMIS Fatigue SFs in RA

### Susan J. Bartlett^1,2^, A. Kristina Gutierrez^2^, Alessandra Butanis^2^, Vivian P. Bykerk^3^, Jeffrey R. Curtis^4^, Amye Leong^5^, Anne Lyddiatt^6^, Ana Maria Orbai^2^, Michelle Jones^2^, W. Benjamin Nowell^7^, and Clifton O. Bingham^2^

#### ^1^McGill University, USA; ^2^Johns Hopkins Medicine, USA; ^3^Hospital for Special Surgery, USA; ^4^University of Alabama at Birmingham, USA; ^5^Healthy Motivation, USA; ^6^Patient Partners, USA; ^7^Global Healthy Living Foundation, USA

**Objective**

In Rheumatoid Arthritis (RA), fatigue is frequent, highly variable, and often severe and disabling. Definitions of fatigue vary, and there is no consensus on how to measure it. We used online surveys and in-person interviews to evaluate the content validity of PROMIS Fatigue short forms (SFs) in people with RA.

**Methods**

We recruited people with RA from an online patient community (n=200) and three academic centers (n=84) in the US. Participants completed SFs then rated the relevance and representativeness of the items to their fatigue experience. Cognitive debriefing of items was conducted in 32 clinic patients. Descriptive statistics were calculated, and associations were evaluated using correlation coefficients.

**Results**

Mean SF scores were similar (p’s≥0.61) among clinic patients reflecting mild fatigue (i.e., 54.5-55.9), but were significantly higher (p’s<.001) in online participants. SF Fatigue scores correlated highly (r’s≥0.82; p’s<.000) and moderately with patient assessments of disease activity (r’s≥0.62; p=.000). Almost all (≥94%) could distinguish general fatigue from RA fatigue. Most (≥85%) rated individual PROMIS items as “somewhat” or “very relevant” to their experience. They averaged their fatigue over the past week (58%), and rated impact vs. severity (72% vs. 19%). 99% rated fatigue as a key indicator of how well their current treatment was controlling their RA.

**Conclusions**

Our results in a large diverse group of adults suggest that items in the PROMIS Fatigue SFs adequately capture the wide range of fatigue experiences of people with RA.

**Keywords**

Fatigue, Validity, PRO, Rheumatoid Arthritis, Outcomes

## P004 Inter-Provider and Problem-Specific Variability of PROMIS-CAT Patient Reported Scores

### Kimberly A. Bartosiak, David L. Bernholt, Jonathan M. Koscso, Amanda G. Spraggs-Hughes, Jennifer Simmons, Christopher M. McAndrew

#### Washington University Orthopaedics, St Louis, MO, USA

**Background**

The objective of this study was to determine if PROMIS-CAT scoring varies between providers or with primary orthopaedic complaint for a given patient.

**Methods**

All patients presenting to an orthopaedic office at this institution complete the PROMIS domains of anxiety, depression, pain interference and physical function. A query was done to collect a group of patients that completed these four domains at two different office visits within one week. Patients were excluded if they were within 6 months from surgical intervention, had an acute trauma or had injection at the first visit as these would be expected to have variability within scores.

**Results**

A control group of patients with two visits with the same provider for the same orthopaedic complaint twice within the same week demonstrated no statistically significant difference between PROMIS scores in any of the four domains collected. A group of patients with two different primary orthopaedic complaints evaluated within the same week by two different providers demonstrated statistically significant differences between visits in anxiety and pain interference domains with improved scores at the second visit. Depression and physical function domains showed no statistically significant differences between visits for any group. For all four domains across all groups more than 20% of patients had scores that differed by at least half of a standard deviation (5 points). This was most profound in the anxiety domain with 38% of patients demonstrating more than a 5 point difference between visits and 13% demonstrating a full standard deviation difference between visits.

**Conclusions**

PROMIS-CAT domains of pain interference and anxiety may be less of a universal reflection of the patient’s status within each domain than domains of physical function and depression which are more consistent across providers and primary complaints.

**Keywords**

PROMIS, Orthopaedic, Anxiety, Depression, Pain Interference, Physical Function

## P005 Can Women Live with More Symptoms than Men? Defining the Patient Acceptable Symptom State (PASS)

### Judith Baumhauer^1^, Adolf Flemister^1^, John Ketz^1^, Benedict Digiovanni^1^, Irvin Oh^1^, Jeff Houck^2^

#### ^1^ University of Rochester Medical Center, Rochester NY, USA; ^2^ George Fox University, Newberg OR, USA

**Objective**

To determine if gender influenced patient reported outcomes specifically patient acceptable symptom state (PASS) and PROMIS PF, PI and Depression in patients with foot and ankle problems.

**Methods**

Prospectively collected PROMIS and PASS were identified for 450 surgical patients (Males=126, Females=324). The CPT code and gender percentages were identified. To assure the overall recovery experienced by females and males was similar aggregate PROMIS scores were compared using ANOVA analysis. The average PROMIS scales were comparable between gender groups with the exception of PROMIS Pain Interference however the difference was not clinically meaningful. The ability of each PROMIS scale to predict PASS status was determined using receiver operator curves (ROC). The area under the curve (AUC) and thresholds for near 95% sensitivity/specificity for males and females for each PROMIS scale. AUC values below 0.7 are not considered clinically useful. Differences AUC or near 95% sensitivity/specificity thresholds by gender would support the hypothesis that PASS status is influenced by gender.

**Results**

There were significant differences in the AUC for gender suggesting PROMIS scores are better predictors of PASS for females than males however there were only minor differences in near 95% sensitivity/specificity PROMIS threshold values by gender. The AUC values for females were higher than for males for each PROMIS scale. The thresholds for PROMIS PF and PI to determine PASS yes status was slightly lower for females (48.5 specificity 94.9) compared to males (52.1 specificity 94.1). The thresholds for PROMIS Dep to determine PASS yes status were similar for females (50.2 specificity 97.0) compared to males (50.6 specificity 94.1). The thresholds for PROMIS PF to determine PASS no status was slightly higher for females (30.0 sensitivity 93.8) compared to males (27.0 specificity 94.4). The thresholds for PROMIS PI to determine PASS no status were slightly lower for females (62.8 sensitivity 93.5) compared to males (68.3 sensitivity 94.4). PROMIS depression thresholds were not considered because of low AUC values.

**Conclusions**

PROMIS scales more accurately predict PASS status in females. In addition, females are more likely to judge their physical abilities as acceptable at a lower PROMIS threshold value compared to males.

**Keywords**

PROMIS, Patient Acceptable Symptom State (PASS), Gender differences

## P006 Predicting Increased Resource Utilization after Carpal Tunnel Release

### Casey Beleckas, William Gerull, Jason Guattery, Charles Goldfarb, Christopher Dy, Ryan Calfee

#### Washington University School of Medicine, USA

**Background**

This study was designed to determine if patient mental health and preoperative experience with pain could predict resource consumption postoperatively.

**Methods**

This retrospective cohort study evaluated all adult patients undergoing isolated unilateral (68%) or bilateral carpal tunnel release (32%) at a tertiary orthopaedic center from 6/1/2015-6/30/2016. All patients completed the PROMIS Pain Interference and Depression Computer Adaptive Testing (CATs) at their pre-operative visit. Postoperative encounters were quantified as a summation of postoperative office visits, phone calls, or electronic messaging related to their carpal tunnel syndrome. Pre-operative opioid use was determined by patient report and prescriptions recorded within 90 days preoperatively. Independent t-tests and chi square testing assessed the differences in initial PROMIS scores between the patients who had one versus more than one postoperative encounter as well as differences in age, sex, race, and opioid use between groups.

**Results**

219 patients who underwent carpal tunnel release were eligible for the study. 59% of patients had a single postoperative encounter while 41% had multiple postoperative encounters (25% had two, 8% had three, and 8% required four or more). Patients who required multiple post-operative encounters had significantly higher pre-operative PROMIS Depression scores (average difference 3 points, 95%CI 0.1-5.5). There was no difference in PROMIS Pain Interference scores or opioid use (each p>0.05). There was also no difference between the groups by unilateral versus bilateral surgery, average age, sex, or race (all p>0.05).

**Conclusions**

While depressive symptoms are thought to influence ultimate patient-reported outcomes, our data now indicate that greater depressive symptoms are also associated with more postoperative encounters after carpal tunnel release. If considering care within a bundled reimbursement model for carpal tunnel syndrome, preoperative PROMIS Depression scores may predict variability in postoperative resource consumption.

**Keywords**

PROMIS, Orthopedic, Resources, Utilization

## P007 Anxiety in the Orthopaedic Patient: Using PROMIS to Assess Mental Health

### Casey Beleckas, BS; Heidi Prather, Jason Guattery, MS; Melissa Wright, Mike Kelly, Ryan P. Calfee

#### Washington University School of Medicine, USA

**Background**

This study explored the performance of the Patient Reported Outcomes Measurement Information System (PROMIS) Anxiety assessment relative to the Depression assessment in orthopedic patients, the relationship between Anxiety with self-reported Physical Function and Pain Interference, and to determine if Anxiety levels varied according to the location of orthopedic conditions.

**Methods**

This cross-sectional evaluation analyzed 14,962 consecutive adult new-patient visits to a tertiary orthopedic practice between 4/1/2016 and 12/31/2016. All patients completed PROMIS Anxiety, Depression, Physical Function, and Pain Interference computer adaptive tests (CATs) as routine clinical intake. Patients were grouped by the orthopedic service providing care and categorized as either affected with Anxiety if scoring >62 based on linkage to the Generalized Anxiety Disorder-7 survey. Spearman correlations between the PROMIS scores were calculated. Bivariate statistics assessed differences in Anxiety and Depression scores between patients of different orthopedic services.

**Results**

Twenty percent of patients scored above the threshold to be considered affected by Anxiety. PROMIS Anxiety scores demonstrated a stronger correlation than Depression scores with Physical Function and Pain Interference scores. Patients with spine conditions reported the highest median Anxiety scores and were more likely to exceed the Anxiety threshold than patients presenting to sports or upper extremity surgeons.

**Conclusions**

One in 5 new orthopedic patients reports Anxiety levels that may warrant intervention. This rate is heightened in patients needing spine care. Patient-reported Physical Function more strongly correlates with PROMIS Anxiety than Depression suggesting that the Anxiety CAT is a valuable addition to assess mental health among orthopedic patients.

**Keywords**

PROMIS, Anxiety, Orthopedic

## O008 Linking FACIT Fatigue to PROMIS^®^ Fatigue Scores in Phase 3 Baricitinib RA Trial Data

### Clifton O. Bingham III^1^, David Cella^2^, Susan J. Bartlett^1,3^, Amy M. DeLozier^4^, Amanda Quebe^4^, Luna Sun^4^, Susan Otawa^4^, Carol Gaich^4^

#### ^1^Johns Hopkins Division of Rheumatology, Baltimore, USA; ^2^ Department of Medical Social Sciences, Feinberg School of Medicine, Northwestern University; Chicago, IL, USA; Departments of Clinical Epidemiology and Rheumatology, McGill University, Montreal, Canada; ^4^Eli Lilly and Company, Indianapolis, IN, USA

**Background**

Fatigue in patients with rheumatoid arthritis (RA) is common and debilitating. In clinical trials, fatigue is often measured with Functional Assessment of Chronic Illness Therapy-Fatigue instrument (FACIT-F; 13-item). The PROMIS Fatigue item bank was developed using a US general population-calibrated T-score metric. PROMIS Fatigue includes the FACIT-F items;scores on the scales are thereby interchangeable. Content debriefing FACIT-F identified 10 items relevant to RA patients. We assessed performance of these items and the 13-item FACIT-F using both crosswalk tables and the PROMIS scoring algorithm on data from 2 phase 3 baricitinib RA trials.

**Methods**

In RA-BEAM, methotrexate-inadequate responders were randomized 3:3:2 to placebo once daily (QD), baricitinib 4 mg QD, or adalimumab 40 mg biweekly (Taylor et al. NEJM.2017;376:652-62). In RA-BEACON, bDMARD-inadequate responders were randomized 1:1:1 to receive placebo or baricitinib 2 mg or 4 mg QD (Genovese et al. NEJM.2016;374:1243-52). Patient-level FACIT-F scores were linked to PROMIS Fatigue scores using validated crosswalk tables (www.prosettastone.org) and the scoring algorithm (http://www.healthmeasures.net/explore-measurement-systems/promis). Analysis of covariance was conducted on PROMIS score conversions to compare responses across treatment arms.

**Results**

At baseline, average PROMIS fatigue scores reflected moderate-to-high levels of fatigue relative to population means, ranging across treatment groups and scoring methods from 57.4 to 59.7 in RA-BEAM and 60.1 to 63.7 in RA-BEACON. Fatigue scores decreased in RA-BEAM to within normal ranges (<55) by week 4 for baricitinib and adalimumab. Statistically and clinically meaningful reductions in mean fatigue scores (exceeding 0.5 SD/5 points) were associated with treatment through 24 weeks in both studies.

**Conclusions**

These results support the FACIT-F to PROMIS Fatigue crosswalk and scoring algorithm approaches, including use of a subset of 10 FACIT-F items deemed most relevant to RA. This enables comparisons across studies that use FACIT-F or PROMIS Fatigue item subsets and their interpretation in US general population.

**Keywords**

Rheumatoid Arthritis; FACIT; PROMIS; Fatigue; Baricitinib

## P009 PROMIS Pain Interference 6b and Fatigue 7a Short Forms and PROFILE-29 in Rheumatoid Arthritis Patients Treated with TNF Inhibitors

### Clifton Bingham III^1^, Shelly Kafka^2^, Shawn Black^2^, Dennis Parenti^2^, Stephen Xu^3^, Wayne Langholff^3^ and Jeffrey R. Curtis^4^

#### ^1^Johns Hopkins University, Baltimore USA; ^2^Janssen Scientific Affairs, LLC. Horsham PA, USA; ^3^Janssen Research & Development, LLC. Spring House PA, USA; ^4^University of Alabama at Birmingham, Birmingham AL, USA

**Background**

PROMIS has been used in rheumatoid arthritis (RA) patients (Pts). AWARE is a multi-center United States-based, real-world evidence study of patients initiating treatment with a Tumor Necrosis Factor inhibitor (TNFi; Simponi Aria or Remicade) in RA and utilizes PROMIS instruments and the Clinical Disease Activity Index (CDAI) to assess effectiveness. This analysis examined selected PROMIS measures to assess (1) relationship between baseline CDAI disease category and T-score, (2) PROMIS responsiveness after initiation of therapy and (3) relationship between T-scores of Profile29v2 Fatigue and Pain Interference questions and respective Short Forms (SF).

**Methods**

We report on TNFi pooled data from 1220 Pts’ baseline PROMIS Pain Interference 6b (PI), Fatigue7a (F), Profile29v2 and CDAI. PROMIS T-scores were compared across CDAI levels of disease activity using ANOVA. We dichotomized pts on baseline T-score: P and F domains T-score >55 vs T-score </=55, and the Physical Function (PF) domain T-score <45 vs T-score >/=45. Data are mean ± standard deviation.

**Results**

Pts were 59.5±13.5 yrs, disease duration 8.2 ± 9.9 yrs, and baseline CDAI score 32.4±15.6. A significant relationship between PROMIS T-scores (PI, F) and baseline CDAI disease activity categories was confirmed. After starting therapy there was minimal change in T-score of pts with baseline PI and F T-scores </=55 and PF>/=45. Pts with baseline PI and F T-scores >55 and baseline PF T-scores <45 showed change from baseline. There was a significant (p<0.0001) relationship between PI and F T-scores and respective 4 questions on the P29v2.

**Conclusions**

We confirm validity of P measures according to CDAI disease category. In RA pts with P T-scores near normal, detecting change once pts start a TNFi therapy may be difficult. Pts with PI, F and PF T-scores >5 units from normal, demonstrated a robust T-score response to therapy.

**Keywords**

Rheumatoid Arthritis, TNF inhibitors, PROMIS-29, Physical Function, Pain Interference, Fatigue

## P010 Identifying Measure Harmonization Needs for the Environmental influences on Child Health Outcomes (ECHO) Program

### Courtney K. Blackwell, Karon F. Cook, Richard Gershon, Dave Cella

#### Feinberg School of Medicine, Northwestern University, Chicago, IL, USA

**Background**

The ECHO Program is a large-scale, transdisciplinary research consortium comprised of 84 extant pediatric cohorts. ECHO’s Person Reported Outcomes (PRO) Core developed a measurement framework for assessing children’s physical, mental and social health outcomes to complement exposure and biological biomarkers. This framework was the foundation for building the ECHO-wide new data collection protocol. An important step in this process was promoting the use of common measures or measures whose scores could be harmonized (e.g., scores could be linked to a common mathematical metric).

**Methods**

A survey was administered to representatives of each of the 84 cohorts. Respondents identified the measures they planned to use to target essential and recommended domains to be assessed in ECHO. The results were collated by domain across cohorts to identify which measures: 1) had already been harmonized (e.g., PROsetta Stone® cross-walks) and 2) would require primary data collection and application of harmonization methodologies.

**Results**

For some target domains, there was consistency in selected measures across cohorts. For other domains, a large number of measures were proposed, only some of which had sufficient evidence of harmonization. Others will require additional investigation to determine the feasibility of score harmonization. As an example, the ECHO Protocol recommends use of the PROMIS Depression scale to evaluate maternal depression, but cohorts proposed using 9 alternative measures, some of which do not have existing PROsetta Stone cross-walks.

**Conclusions**

The results of this study show both the promise and limits of measurement harmonization in large-scale research consortia. The ECHO research program benefits substantially from previous work conducted through PROsetta Stone; but to meet its goals, additional data collection and analyses will be needed to establish more score links to the PROMIS® metric. This study highlights both the recurring benefit of previous score linking efforts and the need for more such studies.

**Keywords**

Child, Patient Reported Outcome Measures, Environment and Public Health, Psychometrics, Scoring Methods

## O011 PROMIS Physical Function Scores Predict Discharge to Home Following Transcatheter and Surgical Aortic Valve Replacement

### Jeffrey Bruckel, MPH^1^, Judith Baumhauer, MPH^2^, Peter A. Knight, ^3^; Craig R. Narins^1^, Chris DaSilva ^3^, Allison W. McIntyre^3^, Charles Lowenstein^1^, Frederick S. Ling^1^

#### ^1^University of Rochester Medical Center, Division of Cardiovascular Medicine; ^2^University of Rochester Medical Center, Department of Orthopedic Surgery; ^3^University of Rochester Medical Center, Division of Cardiac Surgery; ^4^University of Rochester Medical Center, Health Lab PROMIS Program, USA

**Objective**

Aortic Stenosis (AS) is the most common cause of valvular heart disease in the developed world, and is a significant source of morbidity and mortality. Prior to the development of transcatheter aortic valve replacement (TAVR) the only option was surgical replacement (SAVR). Many elderly and functionally limited patients were not candidates for surgery and did not receive treatment. There are several measures of frailty and physical function used to assess patient candidacy for either therapy. We sought to assess the utility of the PROMIS instruments in predicting good treatment outcome following TAVR and SAVR.

**Methods**

We identified a cohort of patients with severe aortic stenosis undergoing evaluation for TAVR or SAVR in multidisciplinary valve clinic. Patients were administered the PROMIS Physical Function (PF), Depression, and Pain Interference (PI) instruments during routine care. We utilized clinical data from the Transcatheter Valve Therapeutics database for TAVR patients, and the Society of Cardiovascular Surgeons database for SAVR patients. Primary outcomes were discharge to home and mortality. Unadjusted analyses were performed using means and t-tests or contingency tables and chi-squared tests as appropriate. Logistic regression was used to obtain risk-adjusted outcomes.

**Results**

The final cohort included 234 patients, 81 patients undergoing SAVR and 135 patients undergoing TAVR. The two groups differed substantially in terms of procedural risk with STS predicted mortality scores of 1.98 (0.80 – 3.17) for SAVR and 7.38 (6.46 – 8.29) for TAVR, p<0.001. TAVR patients were also older, and had higher rates of hypertension, heart failure, prior CABG, atrial fibrillation, cerebrovascular disease, and lung disease (p<0.05 for all). TAVR patients scored significantly lower on PF (33.9 [32.6 - 35.2], p<0.001) and higher on Depression (40.7 [39.1 - 42.4], p<0.001). Patients with higher PF at baseline were more likely to be discharged home (OR 1.12 [1.06 – 1.20] per point increase, p<0.001).

**Conclusion**

PROMIS Physical Function scores are a useful adjunct to clinically-derived measures of frailty, and are predictive of good outcome following TAVR or SAVR.

**Keywords**

Aortic Stenosis, Aortic Valve Replacement, Treatment Selection

## P012 Initial identification of Swedish proxy-report PROMIS^®^ Items for Children Between Four and Eight Years

### John Eric Chaplin^1,2^, Evalill Nilsson^3^

#### ^1^Inst. Clinical Sciences, Sahlgrenska Academy at the University of Gothenburg, Gothenburg, Sweden; ^2^ Swedish Association of Local Authorities and Regions (SALAR), Sweden; ^3^ Dept. of Social Sciences, Department of Medicine and Health, Linköping University, Linköping, Sweden

**Background**

Many paediatric conditions present early in a child’s life. Swedish quality registries wishing to apply PROMIS measures have requested proxy-reported outcome instruments for assessment of children from four years. The aim of this study was to identify proxy-reported items from established PROMIS measures that could be applied to children between the ages of four and eight years.

**Methods**

During a two-day quality review workshop at a Swedish university hospital, 15 health professionals from multiple professions in five teams and two linguistic experts examined 489 items in 19 paediatric item banks. Items considered suitable for proxy report for children under eight years were discussed and agreed within the teams.

**Results**

Forty percent of the items were judged to be suitable for use in the assessment of children from four years of age in proxy report. In total, 196 items were identified as suitable. There was variation within each item bank, ranging from no suitable items in the *Life satisfaction* and *Meaning and purpose* item banks to all items in the *Physical stress* item bank. In nine item banks, the majority of items (>50%) were thought to be suitable for younger children in proxy report.

**Conclusions**

Further work is required in order to confirm the suitability of the questions for younger children; this will be carried out in cognitive debriefing with parents during 2018. Criteria need to be established for the use of these banks in younger children. The calibration of the identified items will be examined to determine whether there is a need for further item development. The next stage of the project is to compare results across languages and to examine the statistical properties of the items. Focus will be on those item banks with greater than 50% of items judged to be appropriate for the younger age group.

**Keywords**

PROMIS, young children, proxy, self-report, Sweden

## P013 The Swedish Linguistic Team-based PROMIS^®^ Item Quality Review Process

### John Eric Chaplin^1,2^, Evalill Nilsson^3^, Elisabeth Ström^4^, Linda Holmström^4^, Rikard Wicksell^4^

#### ^1^Inst. Clinical Sciences, Sahlgrenska Academy at the University of Gothenburg, Gothenburg, Sweden; ^2^ Swedish Association of Local Authorities and Regions (SALAR), Sweden; ^3^ Dept. of Social Sciences, Department of Medicine and Health, Linköping University, Linköping, Sweden; ^4^ Behaviour Medicine, Medical Psychology, Karolinska University Hospital, Stockholm, Sweden

**Background**

To review the linguistic quality of the Swedish translation of child and adult PROMIS item banks.

**Methods**

Multidisciplinary review teams examined the linguistic quality of all Swedish PROMIS item translations. Teams reviewed the reconciliation, forward and backward translations and jointly agreed a final item version. Definitions from the Health Measures organization were used where available, as were item translations from NeuroQoL. Participants were encouraged to ensure that the translation was a conceptual equivalent to the English item and formulated so that it was grammatically and colloquially correct in Swedish.

**Results**

One child-item workshop with a total of five teams and five adult-item workshops with a total of ten teams were held between 2016 and 2018. More than 50 experts from all over Sweden participated, including medical doctors, psychologists, physiotherapists, dieticians, rehabilitation specialists, other health care professionals, patient representatives and linguistic experts. Age and experience varied from full-time professional to head of department. Between one and five teams of three to seven individuals were formed at each workshop. Some experts participated in multiple workshops. Nineteen child-item banks (489 items) and 61 adult-items banks and short forms (781 items) were reviewed. The broad range of professions, nationalities and ages provided a comprehensive view of language usage. Participants quickly learned to work as a team, and gained familiarity with the linguistic requirements of the item formulation. Working on multiple item banks allowed comparison of word usage across banks. Teamwork sustained high levels of motivation throughout. Subjective concepts with multiple equivalent phrasing took longer to agree.

**Conclusions**

Improved item translations were achieved due to this multidisciplinary focus. The methodology and experience gained can be used as an example for other countries interested in translating PROMIS. The Swedish PROMIS items appear linguistically equivalent and ready for cognitive debriefing and cross-cultural validation.

**Keywords**

PRO, Translation, Multidisciplinary, Cross-Cultural, Validation

## O014 Does Recall Period Matter? Comparing PROMIS^®^ Physical Function with No Recall, 24-Hour Recall, and 7-Day Recall

### David M. Condon^1^, Robert Chapman^1^, Sara Shaunfield^1^, Jennifer L. Beaumont^1,2^, Daniel Eek^3^, Debanjali Mitra^4^, Katy Benjamin^5^, Kelly McQuarrie^6^, Jamae Liu^7^, James Shaw^8^, Allison Martin Nguyen^9^, Karen Keating^10^, David Cella^1^

#### ^1^Department of Medical Social Sciences, Feinberg School of Medicine, Northwestern University; ^2^Terasaki Research Institute; ^3^AstraZeneca; ^4^Pfizer; ^5^AbbVie; ^6^Janssen; ^7^Novartis; ^8^Bristol-Myers Squibb; ^9^Merck; ^10^Bayer

**Background**

To evaluate the influence of recall periods on the assessment of physical function, we compared, in cancer and general population samples, the standard administration of PROMIS Physical Function items without a recall period to administrations with 24-hour and 7-day recall periods.

**Methods**

We administered 31 items from the PROMIS Physical Function v2.0 item bank to 2400 respondents (n=1,001 with cancer; n=1,399 from the general population). Respondents were randomly assigned to one of three recall conditions (24-hours, 7-days, or no recall) and one of two “reminder” conditions (with recall periods presented only before the first item or with every item). We tested recall and reminder effects with analysis of variance controlling for demographics, English fluency and comorbidities.

**Results**

Using analysis of variance, each condition was compared to the standard PROMIS administration for Physical Function (no recall period). There was no evidence of significant differences among groups in the cancer sample. In the general population sample, only the 24 hour recall condition with reminders was significantly different from the “no recall” PROMIS standard. At the item level, for both samples, the number of items with non-trivial effect size differences across conditions was minimal

**Conclusions**

For most experimental conditions, when compared to no recall, the use of a recall period has little to no effect upon PROMIS physical function responses or scores. We recommend that PROMIS Physical Function be administered with the standard PROMIS “no recall” period.

**Keywords**

PROMIS, Physical Function, Recall Period, Oncology, Cancer

## O015 Development of PRO T-Score Maps: A New Resource for Clinical Use of PROMIS^®^ Measures

### Karon F. Cook, Nan Rothrock

#### Feinberg School of Medicine, Northwestern University, Chicago, IL, USA

**Background**

A major barrier in the use of PROMIS® measures in clinical settings is the lack of an intuitive score interpretation framework. The objective of this study was to develop PRO T-Score Maps for interpreting PROMIS scores at the item level.

**Methods**

Using an R® program and based on the item parameters of calibrated item banks, we estimated most likely item responses by T-score level for 21 PROMIS item banks. These were used to create labeled heat maps for short form items that graphically display the most likely item responses across the measured score range.

**Results**

On the resulting PRO T-Score Maps, the PROMIS T-score metric is printed horizontally at the top of the page. Items and their response options are displayed in rows below. By tracking item responses with their locations on the T-score metric, users identify T-scores associated with each response across all levels of the domain assessed by the measure. The maps also allow interpretations of the clinical impact of score improvements. For example, for a patient who started with a score of 69 on the PROMIS® Depression measure, a 10 point score decrease would be improving from a probable report of *feeling hopeless* “often” to *feeling hopeless* “rarely”.

**Conclusions**

The PRO T-Score Maps allow users to anchor T-score interpretations within a clinically intuitive context. These maps can be used to interpret both status and change scores and could inform clinical discussions about expectations for improvement and worsening. In the future, PRO T-Score Maps could be developed based on items selected by patients and clinicians. Such maps would anchor score interpretation in items that are most relevant to particular patients or clinical conditions.

**Keywords**

Patient Reported Outcome Measures, Psychometrics, Decision Support Techniques, Score Reporting

## P016 Psychometric Characteristics of a Subset of Items of the PROMIS^®^-MS Fatigue Scale

### Karon F. Cook^1^, Dagmar Amtmann^2^, Paul Kamudoni^3^, Christian Henke^3^

#### ^1^Feinberg School of Medicine, Northwestern University, Chicago, IL, USA; ^2^University of Washington, Department of Rehabilitation Medicine, Seattle, WA, USA; ^3^ Merck, Darmstadt, Germany

**Background**

Previously we developed an 8-item PROMIS® Fatigue short form measuring fatigue in multiple sclerosis (PROMIS-MS-Fat8). A secondary data set that included responses to 6 of the 8 (PROMIS-MS-Fat6) items was analyzed to evaluate reliability and validity in advance of potential use in a clinical trial

**Methods**

N=594 individuals who had MS completed a survey as part of a longitudinal study of outcomes in MS. Available data included Expanded Disability Status Scale (*EDSS*) ratings and self-reported problems with symptoms. Also included were responses to PROMIS Global Health measure and clinical variables. Ten known groups analyses were conducted to compare mean PROMIS-MS-Fat6 T-scores of clinically meaningful groups: a) EDSS: <=4.5, >4.5, b) MS duration: <=5, >5 years, c) PROMIS Global Health, Physical Health, and Fatigue Values: excellent/very good/good vs fair/poor, d) PROMIS Physical Function T-Score of <=median score of 39.8 or > 39.8, 5) MS type: Relapsing Remitting (RRMS) or Progressive (PMS), and e) Spasticity, Imbalance, and f) Bowel/Bladder symptoms: not at all/a little or somewhat/quite a bit/very much. Reliability was assessed by calculating the range of scores in the sample for which reliability was ≥ 0.90. Ceiling and floor effects were defined as endorsing the highest/lowest response to all 6 items.

**Results**

All known groups analyses were statistically significant (p<.001). Reliability ≥ 0.90 was achieved for 91.3% of the full sample and 98.5% of those with T-scores ≥ 50. Ceiling effects in the clinical range of scores were minimal.

**Conclusions**

The results strongly support the validity of PROMIS-MS-Fat6 scores in distinguishing groups expected to have different levels of fatigue. Especially in the clinical range of scores (T-scores ≥ 50), reliability was high and floor/ceiling effects were low. These evaluations should be repeated in a dataset that includes responses to all 8 items of the PROMIS-MS.

**Keywords**

Clinical Trials, Patient Reported Outcome Measures, Multiple Sclerosis, Psychometrics

## O017 First report of patient-reported outcomes in light chain amyloidosis using PROMIS^®^ in a Prospective Interventional Clinical Trial

### Anita D’Souza, MS, Kathryn E Flynn

#### Division of Hematology/Oncology, Department of Medicine, Medical College of Wisconsin, Milwaukee, 53226, WI, USA

**Background**

Light chain amyloidosis (AL) is a rare blood cancer wherein proteins made by malignant plasma cells misfold into amyloid fibrils and deposit in the heart, kidneys, nerves, etc. Patients are often diagnosed late and have high early mortality (30-40%) in the first year after diagnosis. The current standard-of-care using chemotherapy to eradicate the malignancy has no effect on pre-formed fibrils. Patient-reported outcomes (PROs) have not been studied as endpoints in amyloid interventional trials.

**Methods**

In a phase 2 clinical trial studying doxycycline as an anti-fibril agent in in conjunction with chemotherapy (clinicaltrials.gov/NCT02207556), the PROMIS Global Health Index was administered at baseline and monthly intervals during the study period. Patients were staged using the 2012 staging system.

**Results**

31 patients were enrolled including 6 localized and 25 systemic AL. Baseline health was better in localized AL compared to systemic AL (Global Physical Health Score 48.7 vs 42.0; Global Mental Health Score, GMHS 51.3 vs 47.6, respectively). Patients with advanced disease (stage III/IV) had higher GMHS at baseline compared to patients with early disease (stage I/II), 49.1 vs 45.9. Five patients with advanced stage AL died in the first year. In systemic AL, worsening of scores occurred in the first 3-6 months of treatment compared to baseline, likely related to the concurrent use of chemotherapy and known lag time before organ improvement. Both groups showed improvements in PROMIS scores from baseline to end of study.

**Conclusions**

We report longitudinal PROs of AL patients enrolled on an interventional clinical trial. The PROMIS Global Health Index discriminated between groups as hypothesized by stage as well as over time with treatment. Counterintuitively, patients with advanced AL had higher PROMIS GMHS at diagnosis, which may represent relief at receiving a diagnosis for systemic symptoms present for a longer period. This finding needs further exploration.

**Keywords**

PROMIS Global Health Index, AL amyloidosis

## P018 Data Collection Methods in Clinical Setting: A Systematic Review Focusing Time and Ethical Issues

### Walter De Caro ^1^, Elisabetta Corvo ^2^

#### ^1^Sapienza University of Rome, Italy; ^2^Public Health and Health Promotion, Canterbury Christ Church, Canterbury, UK

**Objective**

Nurses are always collecting information (or data) from patients. . Data collected for practice purposes and for research have several key differences. Data collection is a process of collecting information from all the relevant sources to find answers to the research problem, test the hypothesis and evaluate the outcomes.

**Methods**

A systematic review was performed. We reviewed the literature using PubMed, Scopus Google Scholar databases. We selected clinical, health and nursing studies that considered the general population and specific clinical area.

**Results**

19 studies were eligible for inclusion. Data collection techniques were grouped under broad approaches: secondary methods of data collection and primary methods of data collection. At same time we grouped on (1) objective-observation-quantitative (2) subjective-perception-qualitative and (3) physiological-clinical data. We investigate also in consistency and quality of data information and methods for time-consuming reduction and ethical issues.

**Conclusions**

Results from this review suggest that additional research is needed to understand valid methods for collection of data. We have necessity of data collected free from the researchers’ personal biases, beliefs, values, or attitudes.

## P019 Psychometric Properties of some Pediatric Item Banks of PROMIS^®^: Validation in Depressed and Normal Youths

### Inga Dennhag^1^ , John Chaplin ^2,3^, Eva Henje Blom^1^

#### ^1^Child and Adolescent Psychiatry, Department of Clinical Science, Umeå University, Sweden; ^2^Inst. Clinical Sciences, Sahlgrenska Academy at the University of Gothenburg, Gothenburg, Sweden; ^3^Swedish Association of Local Authorities and Regions (SALAR), Sweden

**Background**

Major Depressive Disorder (MDD) in adolescents is a serious risk factor for suicide and future psychiatric co-morbidity. Non-validated, burdensome and inadequate measurement instruments for depression are still widely used in clinical practice. Previous research suggests that the pediatric Patient-Report Outcomes Measurement Information System (PROMIS) has potential to meet the challenges of measurement in an adolescent population with depression, however, no reference data exists from a Swedish population. The purpose of this study is to validate and evaluate thirteen pediatric PROMIS item banks in a population of 12-19 year olds.

**Methods**

A cross-sectional analysis will be conducted on a non-clinical population of >600 adolescents drawn from junior and high-schools in northern Sweden. PROMIS Item banks translated into Swedish following the FACIT-trans method and the Swedish review workshop procedure will be used. The banks measure depression, anxiety, anger, positive affect, psychological stress, cognitive function, life satisfaction, meaning and purpose, fatigue, physical activity, pain interference, peer and family relationships.

**Results**

Ethics approval for the study has been given and data collection will start autumn 2018**.** We will use differential item functioning (DIF) analysis to evaluate parameter stability between the Swedish adolescent population and the US population; between genders and by age; we will also examine residual correlations to evaluate local dependence among Swedish items to compare against the US. Where possible correlation with other scales will be undertaken. The approach to the contact with schools and the use of an informed consent procedure with the parents will be described.

**Conclusions**

The identification and evaluation of item banks that can be used in a psychiatric population to measure constructs of health-related quality-of-life is a fundamental objective of this project. Validated item banks will be used as the foundation for developing short-form instruments and in the future enabling computerized adaptive testing.

**Keywords**

PROMIS, depression, screening, computerized adaptive testing, Differential item functioning

## P020 Validation of a method to generate PROMIS^®^-Preference (PROPr) Scores when PROMIS cognitive function scores are missing in a population of community cancer patients

### Barry Dewitt^1^, Roxanne E. Jensen ^2^, Janel Hanmer^3^

#### ^1^Department of Engineering & Public Policy, Carnegie Mellon University, Pittsburgh, PA; ^2^Leidos Biomedical Research contract, supporting the National Cancer Institute; ^3^Division of General Internal Medicine, University of Pittsburgh, Pittsburgh, PA, USA

**Background**

The PROMIS-Preference (PROPr) scoring system produces a societal-preference based summary score from 7 PROMIS domains: Cognitive Function (v2), Depression, Fatigue, Pain Interference, Physical Function, Sleep Disturbance, and Ability to Participate in Social Roles and Activities. Many studies using PROMIS to date use a PROMIS Profile, which does not include cognition function (e.g., the PROMIS-29). Our objective is to validate a method for estimating PROPr scores without cognitive function scores, using a dataset of cancer patients.

**Methods**

The Measuring Your Health Study administered all 7 PROMIS domains used in PROPr in a community-based sample (n=5506) of patients diagnosed with cancer, including a 6-month follow-up (n=2968, follow-up rate: 54%). We evaluated a linear regression model for predicting PROPr scores when cognitive function is missing by estimating the generalization error for patients at baseline, follow-up, as well as the ability to recover changes in the PROPr score for those measured at both baseline and follow-up. We evaluated out-of-sample prediction via root-mean-squared-error (RMSE) and mean error (ME).

**Results**

The RMSE (ME) for predicting PROPr scores at baseline is 0.056 (-0.013), on PROPr’s -0.022 to 1 scale. The RMSE (ME) for predicting PROPr scores at follow-up is 0.059 (-0.014). The RMSE (ME) for predicting changes in the PROPr score is 0.060 (-0.0011).

**Conclusions**

PROPr, a societal preference-based summary score, can be generated for data that are missing measurements on the cognitive function PROMIS domain, such as datasets that include the PROMIS-29.

**Keywords**

PROMIS-Profile, Health Utility, Propr, Cancer

## P021 Differentiating Coping Behaviors in Predicting NIH Toolbox Psychological Well-Being

### Elizabeth M. Dworak (Knowlton)^1^, Robert Chapman^2^, and David M. Condon^2^

#### ^1^Department of Psychology, Northwestern University, Evanston, IL, USA; ^2^Department of Medical Social Sciences, Northwestern University, Chicago, IL, USA

**Background**

Existing researched has emphasized how coping strategies effect psychological well-being, but studies have failed to examine which specific coping behaviors are associated with high or low psychological well-being. In this study, we investigate the relationship between NIH Toolbox measures of psychological well-being and endorsement of primary coping behaviors in a large international internet-based sample. We hypothesized that greater psychological well-being would be associated with adaptive coping behaviors and that lower psychological well-being would be associated with maladaptive coping behaviors.

**Methods**

Utilizing a large web-based sample, we collected data from (N=26,770), participants through the data collection platform at SAPA-Project.org. Each participant endorsed one of nine coping behaviors, responded to subsets of items from two NIH Toolbox scales of Psychological Well-Being (General Life Satisfaction & Meaning and Purpose). To test our hypotheses, we analyzed the resulting dataset with tetrachoric and polychoric correlations between variables and scales.

**Results**

In testing our main hypothesis, we found that lower psychological well-being was associated with maladaptive coping behaviors, such that both General Life Satisfaction & Meaning and Purpose showed a negative or zero association with eating (r=-0.40, r=-0.14), substance use (r=-0.41, r=-0.57), ignoring stress (r=-0.02, r=-0.17), and distraction (r=-0.20, r=0.00). Additionally, high psychological well-being was associated with adaptive coping behaviors such that General Life Satisfaction & Meaning and Purpose showed a positive association with exercise (r=0.48, r=0.44), meditation/mindfulness (r=0.51, r=0.51), and spiritual practice (r=0.51, r=0.17).

**Conclusions**

Psychological well-being is an important construct that is associated with the selection of one’s primary coping behavior. The utilization of adaptive or maladaptive coping behaviors can skew one’s self report of General Life Satisfaction & Meaning and Purpose in life. The interplay between these psychological constructs should be further explored to elucidate if the association between lower psychological well-being in higher at-risk demographics can further predict maladaptive coping behaviors.

**Keywords**

Coping, Psychological Well-Being, Mental Health, Behavior, Coping Behavior, NIH Toolbox, SAPA (Synthetic Aperture Personality Assessment)

## O022 PROMIS^®^ Domains Explain a Large Proportion of Quality of Life in Patients with Advanced Chronic Kidney Disease

### Oladapo Ekundayo^1^, Nathaniel Edwards^1^, Aarushi Bansal^1^, Abeera Ali^1^, Evan Tang^1^, Allen Xu ^1^, Punithan Thiagalingam^1^, Marta Novak^2^, Istvan Mucsi^1^

#### ^1^Multi-Organ Transplant Program and Division of Nephrology, University Health Network, Toronto, Canada; ^2^Centre for Mental Health, University Health Network, Toronto, Canada

**Background**

Quality of life is a complex construct influenced by sociodemographic, clinical and psycho-social factors. The Patient Reported Outcomes Measurement Information System (PROMIS) project has developed generalizable and universal PROMs. There has been little research to assess the relative contribution of clinical, socio-demographic and PROM variables to explaining health related quality of life (HRQOL) in advanced chronic kidney disease (CKD). Here we assess if adding PROMIS® domains will increase the explanatory power of models predicting HRQOL of patients with advanced CKD

**Methods**

This cross-sectional cohort study involved patients with advanced CKD (dialysis and post-transplant) recruited from two hospitals in Toronto. The depression, physical function, pain, sleep and fatigue domains of the PROMIS-57 questionnaire were completed electronically. Socio-demographic and clinical variables were collected from medical records. The EuroQoL (ED-5Q-5L) was used to measure of HRQOL. Linear regression models were fitted with expanding sets of co-variables to assess the contribution of the PROMIS domains on predicting HRQOL.

**Results**

Mean (SD) age of the 339 patients was 56 (17) years with 58% males and 50% Caucasians. The ED-5Q-5L scores ranged from 0.12 to 0.9. The model that included socio-demographic variables only (age, gender, ethnicity, marital status, education, income) explained only 5% of the variance (adjusted R^2^=0.05). When clinical factors (renal replacement modality, comorbidities, hemoglobin and albumin levels) were added, the adjusted R^2^ was 0.17. Adding PROMIS domains (pain, physical function, depression, sleep and fatigue) increased variance prediction to 63% (Adjusted R^2^ = 0.63). Predicted values from the final model showed strong correlation with measured EQ-5D-5L scores (r=0.805, p<0.001).

**Conclusions**

The PROMIS domains provide important information about HRQOL in patients with advanced CKD . Further research is needed to assess if the PROMIS domains predict additional outcomes (eg. mortality) above and beyond clinical and socio-demographic variables.

**Keywords**

PROMIS; quality of life; chronic kidney disease

## P023 A Cross-Sectional Follow-Up Study of a Two-Historical Childhood Bacterial Meningitis Cohorts on Long-Term Outcome

### O El Tahir^1,2^, RCJ de Jonge^3^, S Ouburg^2^, SA Morre^2,4^, AM van Furth^1^

#### ^1^Department of Pediatric Infectious Diseases, VU University medical center, Amsterdam, The Netherlands; ^2^Department of Medical Microbiology and Infection Control, Laboratory of Immunogenetics VU University Medical Center, Amsterdam, The Netherlands; ^3^Department of Pediatrics, Division of Neonatology, VU University medical center, Amsterdam, The Netherlands, ^4^Department of Genetica and Cell Biology, Institute for Public Health Genomics (IPHG), Research School GROW (School for Oncology & Developmental Biology), Faculty of Health, Medicine & Life Sciences, University of Maastricht, The Netherlands

**Background**

This follow-up study aims to provide more insight into quality of life childhood bacterial meningitis (BM) could have in later life. BM is a serious, life-threatening infectious disease of the central nervous system (CNS) that often occurs in young children. Approximately one-fifth of the children who survive BM are left with sequelae. Considering sequelae, it is largely unknown whether these sequelae persist in adolescence and adulthood.

**Methods**

Adolescents and young adults (n = 947) are invited to determine health- related quality of life using PROMIS Global Health 10, PROMIS-29 Profile and PROMIS Satisfaction 2.0 questionnaires. Participants of the present study originally belong to two historical cohorts of childhood BM survivors selected in 1999 and 2005. All participants consented on approach during follow-up studies. As most of the questionnaires are validated the results will be compared to norm data by one-sample t-tests.

**Results**

Data of 464 survivors were analyzed. Mean age of the sample was 25.3 years (range 18.0-32.0). Survivors scored significantly lower on all PROMIS-29 Profile scales and the PROMIS Satisfaction 2.0 questionnaire.

**Conclusions**

BM during childhood seriously impacts quality of life during adolescence and adulthood.

**Keywords**

Bacterial meningitis, quality of life, PROMIS, children

## O024 Identifying Foot and Ankle Patients at Risk to Fall Based on Patient Reported Outcome Assessments

### Kathleen Fear^1^, Jack Teitel^1^, Christopher Dasilva^1^, Allison McIntyre^1^, Jeff Houck^2^, David Mitten^1^, Judith Baumhauer^1^

#### ^1^ University of Rochester Medical Center, Rochester NY; ^2^ George Fox University, Newberg OR, USA

**Background**

Each year approximately 30-40% of people over the age of 65 fall. Approximately one half of these falls result in an injury with the estimated annual direct medical costs of $30 billion. Identifying patients at risk to fall and implementing a prevention plan would help patients and save cost to the healthcare system.

**Methods**

Prospective PROMIS CAT physical function, pain interference, depression, fall risk assessment questions and patient demographics were collected for all patients at each clinic visit from an academic orthopaedic multi-specialty practice between January 2015 and November 2017. Standardized yes/no validated self-reported fall risk questions include: “Have you fallen in the last year?” and “Do you feel you are at risk of falling?” Histograms, t-tests, confidence intervals and effect size were used to determine the fall risk “YES” patients were different than the “NO” for ALL orthopaedic patients and specifically foot and ankle patients. Logistic Regression was used to determine if age, gender, height, weight, and PROMIS scales predicted self-reported falls risk.

**Results**

94,761 orthopaedic patients comprising 315,273 visits (44% male, mean age 53.7+/-17 years) and 13,720 foot/ankle patients comprising 33,480 visits (37% male, mean age 52.7+/-16.1 years) had complete data for analysis. Although all PROMIS scores demonstrated significant impairment between patients at risk designation (yes/no), PROMIS PF had the largest effect size for ALL Ortho and FOOT AND ANKLE patients (0.8 and 0.7 respectively). Patients who are at risk to fall have PROMIS PF t-scores >1.5 lower than the United States normative population while the patients not at risk are less <1 SD. In the adjusted regression models gender and PROMIS PF had the largest coefficients.

**Conclusions**

PROMIS PF t-scores of <35 can be linked to self-reported fall risk.

**Keywords**

Fall Risk, PROMIS, Patient-Reported Outcomes

## O025 Validation of Dutch-Flemish PROMIS® Computerized Adaptive Tests for Depression and Anxiety

### Gerard Flens^a^, Niels Smits^b^, Caroline Terwee^c^, Philip Spinhoven^d^, Edwin de Beurs^a^

#### ^a^ Foundation for Benchmarking Mental Health Care, Bilthoven, The Netherlands; ^b^ Research Institute of Child Development and Education, University of Amsterdam, The Netherlands ^c^ Department of Epidemiology and Biostatistics, The EMGO Institute for Health and Care Research, VU University Medical Center, Amsterdam, The Netherlands; ^d^ Department of Psychiatry, Institute of Psychology, Leiden University, The Netherlands

**Objective**

Many instruments that are used in mental health care are either inefficient and precise or efficient and imprecise, as they are based on classical test theory. To overcome this problem (among others), the Unites States PROMIS initiative has developed a set of instruments based on item response theory (IRT), using computerized adaptive testing (CAT). In the Netherlands, Dutch-Flemish CATs are validated using the PROMIS adult V1.0 item banks for Depression and Anxiety. The validation aspects concern both single measures and longitudinal measures, as well as comparisons of both groups and individuals.

**Methods**

First, cross-sectional data of the full item banks (N = 2010) was psychometrically evaluated using IRT (i.e., the Graded Response model; GRM) and Structural Equation Modelling (SEM). Second, data of the full item banks became recently available to evaluate longitudinal measurement invariance of the item banks using SEM (N = 500). Third, data is being collected to evaluate responsiveness of the CATs.

**Results**

The evaluation for cross-sectional use indicates excellent psychometric properties of the item banks. Furthermore, both item banks showed efficient and highly precise measurement applying a CAT simulation, and a similar accuracy between this CAT simulation and the full item bank administration. For the two studies concerning longitudinal measurement, we will present (preliminary) findings.

**Conclusions**

PROMIS offers assessment of patient-reported mental health - with an internationally applicable assessment battery - that is more efficient and precise than existing PROMs. Regarding measurement invariance and responsiveness, results may show that the Dutch-Flemish CATs are also superior to existing PROMs.

**Keywords**

Anxiety, Depression, Psychometric Properties, Computerized Adaptive Testing

## P026 Validating Machine Learning Prediction of Minimally Clinically Important Changes in PROMs After Total Joint Arthroplasty

### Mark Alan Fontana^1,2^, Douglas E. Padgett ^1^, Catherine H. MacLean^1^

#### ^1^Center for the Advancement of Value in Musculoskeletal Care, Hospital for Special Surgery, New York, NY, USA; ^2^Department of Healthcare Policy and Research, Weill Cornell Medical College, Cornell University, New York, NY, USA

**Background**

Identifying patients at risk of not achieving minimally clinically important changes (MCICs) in PROMs after total joint arthroplasty (TJA) is important for better allocating resources toward monitoring patients and may aid in decision support. However, the ability of such predictive models to work across different PROMs, data sources, and time horizons is unknown.

**Methods**

We applied a machine learning (ML) algorithm, logistic LASSO, to hip and knee registry data from a high-volume facility to predict 2-year MCICs in SF-36 physical (PCSs) and mental component scores (MCSs). We derived models that incrementally incorporated information available: (1) before the decision to have surgery, (2) before surgery, (3) before discharge, and (4) after discharge. We evaluated performance with area under the receiver operating characteristic (AUROC) statistics using a hold-out sample of registry patients not used in model creation. We further tested whether these models could predict 6-month MCICs in PROMIS-10 PCSs and MCSs in a validation sample from our EMR.

**Results**

12,203 registry patients had valid baseline and 2-year scores. AUROCs for predicting 2-year SF-36 PCS MCICs at the four time points were: 0.67, 0.74, 0.74, and 0.75. For MCS MCICs these were: 0.54, 0.88, 0.88, and 0.88. The EMR validation sample included 1,087 patients. Reusing the registry models, AUROCs for predicting patients’ 6-month PROMIS-10 PCS MCICs at the four time points were: 0.56, 0.63, 0.63, and 0.65. For MCS MCICs these were: 0.50, 0.78, 0.78, and 0.79.

**Conclusions**

ML algorithms applied to registry data can predict 2-year post-surgical SF-36 PCS and MCS MCICs. Applying these models to EMR data to predict 6-month PROMIS-10 MCICs retains some, but not all, of their predictive power. Across PROMs, data sources, and time horizons, information available before surgery, namely baseline PROMs, yielded the largest gain in predictive power; including available post-surgical information yielded negligible improvement.

**Keywords**

Machine Learning, Prediction, Proms, Minimally Clinically Important Change, MCIC, Total Joint Replacement, Total Joint Arthroplasty, Hip, Knee, Robustness, Validation, AUROC

## O027 Validity and Clinically Important Differences on the PROMIS^®^ Physical Function Short Form in Parkinson Disease

### Ann L. Gruber-Baldini^1^, Erik Barr^1^, Lisa M. Shulman^2^

#### ^1^Department of Epidemiology & Public Health, University of Maryland School of Medicine, USA; ^2^Department of Neurology, University of Maryland School of Medicine, USA

**Background**

The goal was to examine the validity and clinically important differences on the PROMIS-Physical Function (PROMIS-PF) in Parkinson Disease (PD) patients.

**Methods**

Cross-sectional and one-year (12+/- 6 months) longitudinal data were collected on subjects from the University of Maryland PD Center research database. Questionnaires completed by PD patients included PROMIS-PF(Short Form 4a), Older Americans Resource and Services Activities of Daily Living (OARS ADLs), and falls in last two weeks. Physicians completed Unified Parkinson’s Disease Rating Scale (UPDRS) total and motor, Schwab and England (S&E) ADL scale, and Hoehn and Yahr (HY) stage. Correlations and tests of Clinically Important Differences derived from distribution-based (per standard deviation) and anchor-based (per meaningful cutpoints on other scales) approaches were examined.

**Results**

Data were available from 849 cross-sectional and 422 longitudinal patients. Patients were predominantly male (63%), white (90%), nonfallers (72.1%), an average age 67.7±9.8, Montreal Cognitive Assessment 24.4±4.8, PD duration 7.7±6.5 years, HY 2.8±1.1, UPDRS total 36.7±18.2, UPDRS motor 24.3±11.3, S&E 79.7±14.9, OARS ADL 21.4±9.3 and PROMIS-PF 45.3±9.8.

PROMIS-PF was significantly (p<.001) correlated with OARS (r=-0.82), S&E (r=0.65), UPDRS total (r=-0.62), and motor (r=-0.53) cross-sectionally, but changes over time correlated less: S&E r=0.21, p=.001; UPDRS total r=-0.17, p=.006; motor r=-0.15, p=.001; OARS r=0.01, p=.94.

A minimally important difference of 3 t-score units on PROMIS-PF corresponded cross-sectionally with differences across every HY stage and between fallers and nonfallers, and longitudinally with clinically meaningful declines on UPDRS total and motor scales. Differences over time among improvers on UPDRS were smaller than for decliners.

**Conclusions**

In a sample of PD patients, the PROMIS-PF had good concurrent validity in that it correlated well with other patient- and physician-rated scales cross-sectionally and longitudinally. A difference or change of 3 t-score units was able to distinguish between most clinically meaningful groups, especially with regard to decline or worse functioning.

**Keywords**

PROMIS Physical Functioning, Parkinson Disease, Validity, Clinically Meaningful Difference, Activities Of Daily Living

## P028 Implementing and Integrating Patient Reported Outcomes Data Capture in an Academic Medical Center Setting

### Jason Guattery^2^, MS, Amanda Spraggs-Hughes^1^, MA, Ryan P. Calfee^1^

#### ^1^Washington University in St. Louis, USA; ^2^University of Pittsburgh, USA

**Background**

The primary aim was to develop department-wide electronic data capture (EDC) of Patient Reported Outcome (PRO) measures at all ambulatory Orthopaedic clinical visits through a custom developed web-based application (WUPRO) that was minimally disruptive to existing clinical workflows. Secondary aims included the further integration of WUPRO with existing information technologies to support clinical and research applications reliant on PRO data.

**Methods**

A project manager and clinical administration developed a minimally invasive workflow for EDC. A cross-sectional team was brought together to review and help address identified barriers to implementation throughout the process.

Technical development worked with an iterative approach that built, refined, and customized functionality throughout the implementation process. Daily meetings were used to address barriers and refine potential technical solutions.

Successful implementation was measured through the use of administrative reports. Amongst data reports, capture rate and completion rate were the primary markers for success.

**Results**

Implementation of PRO data capture via WUPRO was successfully achieved over a period of six months (6/22/15 – 12/16/15). Department-wide capture rate at the conclusion of the implementation pilot was 99% and the completion rate was 99%. The patient population was generally accepting of the EDC system, with our patient population refusing to complete the assessments at 1.4% of visits. Two years after the initial pilot the department has maintained a capture rate of 99% and a completion rate of 95%. The refusal rate at visits is 1.2%.

**Conclusions**

Our implementation success was dependent on multiple factors including buy-in across all levels of the department, development of a flexible EDC system, and a collection process with a minimal footprint. Multidisciplinary meetings to go over implementation concerns as well as regular monitoring of staff performance provided support and allowed the implementation group to identify and address issues before they became significant barriers to data collection.

**Keywords**

PROMIS, Patient Reported Outcomes, Orthopaedic Surgery

## P029 Lessons Learned and Future Directions for Patient Reported Outcome Usage at an Academic Medical Center

### Jason Guattery^2^, Amanda Spraggs-Hughes^1^, Ryan P. Calfee^1^

#### ^1^Washington University in St. Louis, USA; ^2^University of Pittsburgh, USA

**Objective**

To describe the lessons learned from the implementation and early use of PRO data collection in the orthopaedic surgery department of an academic medical center.

**Methods**

The orthopaedic surgery department served as the pilot department for outpatient PRO collection. The implementation team introduced PRO data collection at 7 different clinical sites and each new launch allowed the team to refine technical and logistical procedures. Major technical obstacles were identified addressed by the implementation team prior to launch and minor obstacles were largely eliminated by the time full-scale departmental delivery of PRO assessments had begun. The implementation team, alongside appropriate clinical stakeholders, addressed any unforeseen issues that arose. Lessons learned were applied to future implementation sites.

**Results**

Provider and patient adoption of PRO data collection were among the most difficult hurdles to overcome for our department. Feedback received from clinical faculty and staff suggest that enhanced educational offerings would assist in preparing for implementation. Our patient populations provided feedback on specific modules (depression, anxiety) that indicated a lack of understanding how mental health impacts clinical healing.

**Conclusions**

Adoption of PRO assessment collection at our institution required significant support, both among our institutional executive committee and department level leadership. Despite this support, the major findings from our institutional pilots suggest that even more robust education for physicians, clinical support staff, and patients prior to implementation of PRO data collection is necessary in order to ensure smoother adoption. Technical hurdles offer another area of improvement, yet will vary by institution based on systems already in place and available resources.

Our institution is in the process of deploying PRO collection in additional departments throughout the medical center. Critical lessons learned in the orthopaedic department were integral to honing the process of implementation and will assist future practitioners utilizing PRO data at our institution.

**Keywords**

PROMIS, patient reported outcomes, orthopaedic surgery

## P030 Cross-Cultural And Construct Validity of the Dutch-Flemish PROMIS^®^ Upper-Extremity Item Bank v2.0

### Erik-Jan A. Haan^1,2,5^, Caroline B. Terwee^2^, Marieke F. van Wier ^3^, Derek F.P. van Deurzen^3^, Nienke W. Willigenburg ^3^, Martijn F. Pisters^1^, Aaron J. Kaat ^4^, Leo D. Roorda ^5^

#### ^1^Physical Therapy Sciences, Program in Clinical Health Sciences, University Medical Center Utrecht, Utrecht, The Netherlands; ^2^Department of Epidemiology and Biostatistics, Amsterdam Public Health Research Institute, Amsterdam UMC, location VUmc, Amsterdam, The Netherlands; ^3^Department of Orthopedic Surgery, Joint Research, OLVG, Amsterdam, The Netherlands; ^4^Department of Medical Social Sciences, Feinberg School of Medicine, Northwestern University, Chicago, Illinois, USA; ^5^Amsterdam Rehabilitation Research Center | Reade, Amsterdam, The Netherlands

**Background**

The aim of this study was to examine the cross-cultural and construct validity of the Dutch-Flemish PROMIS^®^ Upper-extremity (UE) item bank v2.0 in a Dutch population of patients with musculoskeletal upper-extremity disorders.

**Methods**

Four PROMIS v2.0 physical function items, measuring upper-extremity function, were translated into Dutch-Flemish. These items were combined with 42 already translated items, to develop the 46-item Dutch-Flemish PROMIS UE item bank v2.0. To examine validity, a cross-sectional design was used. Two hundred five patients referred to the Orthopedic Department of a general hospital in The Netherlands, aged 18 years or older, with musculoskeletal disorders of the upper extremity, were included between February and May 2018. Participants filled in a questionnaire with demographic and clinical characteristics, the Dutch-Flemish PROMIS UE item bank v2.0, and four legacy instruments. For cross cultural validity, Differential Item Functioning (DIF) was evaluated for language. For construct validity, a-priory hypotheses were tested for correlations with the legacy instruments. DIF was evaluated by ordinal logistic regression models. When items were flagged as potential DIF for language items, the impact of DIF was examined by plotting item characteristic curves and test characteristic curves. Correlations were quantified by Pearson’s or Spearman’s correlation coefficient.

**Results**

Eight items showed minimal DIF for language which resulted in sufficient cross-cultural validity. The Dutch-Flemish PROMIS UE item bank had a moderate correlation with the Dutch-Flemish PROMIS Pain Intensity item (r = -0.43) and strong correlations with the Disabilities of Arm, Shoulder and Hand Questionnaire (r = -0.87), the Functional Index of Hand OsteoArthritis (r = -0.86) and the Michigan Hand Outcomes Questionnaire (r = 0.81), all correlations were as hypothesized.

**Conclusions**

The Dutch-Flemish PROMIS UE item bank v2.0 has sufficient cross-cultural validity and construct validity.

**Keywords**

Patient-Reported Outcomes Measurement Information System, PROMIS, Upper-extremity, Validation

## O031 Cross-Sectional Validity of the PROMIS^®^-Preference (PROPr) Score for Population Health Measurement

### Janel Hanmer^1^, Mark Roberts^2^, Paul Pilkonis ^3^

#### ^1^Division of General Internal Medicine, University of Pittsburgh, Pittsburgh, USA; ^2^Department of Health Policy and Management, University of Pittsburgh Graduate School of Public Health, Pittsburgh, USA; ^3^Department of Psychiatry, University of Pittsburgh, Pittsburgh, USA

**Objective**

To evaluate if the PROPr score is associated with social determinants of health.

**Methods**

Respondents were from a US general population panel ages 18 and older maintained by NORC. Respondents could complete the survey online or by phone, in English or Spanish. The survey included the 7 PROMIS domains necessary to calculate PROPr (Cognitive Function—Abilities, Depression, Fatigue, Pain Interference, Physical Function, Ability to Participate in Social Roles and Activities, and Sleep Disturbance), 12 self-reported health conditions, and self-reported social determinants of health. Household location was linked to census tract data. A condition or social determinant impact estimate was created using ordinary least squares regression in which PROPr was regressed on age, gender, and the health condition or social determinant.  Analyses were weighted to be nationally representative.

**Results**

There were 4142 completed surveys. PROPr scores (n=4114) ranged from -0.02 to 1.0 with a mean of 0.49. For all 12 health conditions, the age- and gender-adjusted PROPr scores were statistically significantly lower for those who reported the condition compared to those who did not report the condition. The smallest impact was for -0.04 cancer (p<0.01) and the largest impact was -0.23 for emphysema (p<0.0001). Many self-reported social determinants of health were also associated with PROPr scores. For example, there were statistically significant estimates for those who were uninsured (-0.04, p<0.01), food insecure (-0.21, p<0.0001), or reported little social support (-0.13, p<0.0001).  Some census tract level variables were also associated with PROPr scores: being in a tract with the highest quartile of poverty (-0.04, p<0.0001), highest use of food stamps (-0.06, p<0.0001), or highest unemployment rate (-0.06, p=0.002).

**Conclusions**

The PROPr score is associated with both chronic health conditions and social determinants of health.  These results provide cross-sectional validity for the use of the PROPr score for population health measurement.

**Keywords**

PROMIS, Health Utility, Propr, Validation, US General Population

## P032 A Protocol for the Validation of the Swedish Version of the PROMIS^®^ Global Health (GH) Scales in Adults and Children

### Linda Holmström^1^, Rikard Wicksell^1^, Elisabeth Ström^1^, Evalill Nilsson^2^, John Eric Chaplin^3,4^

#### ^1^ Behaviour Medicine, Medical Psychology, Karolinska University Hospital, Stockholm, Sweden; ^2^ Linköping University, Dept. of Social Sciences, Department of Medicine and Health, Linköping University; ^3^ Inst. Clinical Sciences, Sahlgrenska Academy at the University of Gothenburg, Gothenburg, Sweden; 4 Swedish Association of Local Authorities and Regions (SALAR), Sweden

**Background**

To outline a protocol for the validation of the Swedish versions of the PROMIS global health scales and initiate the implementation of the scales in multiple clinical environments.

**Methods**

Adults and children and parents attending the Karolinska hospital together with a school-based sample of ‘healthy normal’ children will use an electronic data collection system to complete the GH-10 + EQ5D and the GH-9 + EQ5DY. Demographic and clinical data will be collected by an electronic records system (Take Care). Analyses will include the internal consistency and factor analysis. Reliability following a two-week test-retest procedure will be assessed. A differential function analysis will investigate if the items show signs of interaction with sample characteristics. Discriminant ability will be evaluated via known groups’ analysis and responsiveness via before and after treatment evaluation.

**Results**

It is planned that a sample of 500 adults and 500 children/parents will be surveyed starting in 2019. The results of the study will indicate the statistical validity of the instruments and their clinical value in routine care. It is anticipated that the study will be able to identify different response patterns across diagnoses, gender, and age that will assist in the treatment process.

**Conclusions**

Before implementing the global health scales satisfactory statistical characteristics must be demonstrated; and they must be shown to be acceptable by both adults and children. Responsive to changes in condition and treatment change will be an important factor in the acceptance of the instrument for clinical routine use. However, it is vital that a methodology is identified for the implementation of the instruments within the clinical setting so that the results are used in routine clinical judgement. Key clinical sites within the hospital able to demonstrate clinical use will be targeted for the validation study thus encouraging hospital wide implementation.

**Keywords**

PROMIS, Generic measures, adults, children, global health

## P033 Influence of Socioeconomic Factors and Patient Reported Outcome Measurement Information System on Patient Acceptable Symptom State (PASS)

### Jeff Houck^1,2^, Kiah Mayo^2^, Chris Dasilva^2^, Judy Baumahuer^2^

#### ^1^George Fox University, Newberg, OR; ^2^University of Rochester, Rochester, NY, USA

**Background**

To evaluate the relative contributions of socioeconomic variables (age, gender, income, and race) and Patient Reported Outcome Measurement Information System (PROMIS) health domains (physical Function (PF), pain interference (PI), and depression (Dep) on predicting patient acceptable symptom state (PASS) in patients with foot and ankle problems.

**Methods**

From a large data base 5499 unique patients with complete PASS and PROMIS data were identified. A total of 30.2% of patients attending a foot and ankle orthopedic service on the first available visit identified as PASS yes. Geocoding was used to estimate median income based on the 2010 US census. The sample average age was 52.3±16.4. The proportion of females was 63.6%; race was 85.9% White, 10.3% Black, and 1.9% were Asian and 2.9% other. Median income categories varied from Federal Poverty level(FPL) (<$24,999) to Upper Middle Class or higher (UMCOH) (>100K). PROMIS variables were converted to dichotomous variables using receiver operator curve analysis (PF>42, PI<56.2, Dep<47.8). Logistic regression models were explored to determine odds ratios (OR) for the best model to predict PASS.

**Results**

The highest OR was for PROMIS PI (4.02 95% confidence interval (CI) 3.45-4.67). The PROMIS Dep scale was also significant (1.31 95% CI 1.14-1.50), however, PROMIS PF was not significant (p=0.13). Income (0.88 95% CI 0.83-0.93) and race (1.15 95%CI 1.03-1.27) were also significant while gender (p=0.28) and age(p=0.17) were not.

**Conclusions**

The strongest predictor of PASS across variables was pain interference. While several other variables were also independent predictors of PASS, and significant, their OR were relatively close to 1, suggesting low clinical significance. Clinicians should consider PROMIS PI and Dep as better indicators of PASS than socioeconomic variables.

**Keywords**

Socioeconomic, patient acceptable symptom state, PROMIS

## P034 PROMIS^®^ Scales are Predictive of Patient Acceptable Symptom State in Primary Care

### Jeff Houck^1^, Dan Kang^1^, Tyler Cuddeford^1^, Sara Rahkola^2^

#### ^1^George Fox University, Newberg, OR Primary Medical Group, USA; ^2^Providence Health Systems, Newberg, OR, USA

**Background**

To assess the ability of PROMIS health domains (physical function (PF), pain interference (PI), self-efficacy of symptom management (SE) and activity limitations (SEAD)) to determine patients that are at an acceptable symptom and activity level (PASS).

**Methods**

From Dec 2016 to Aug 2017 102 patients were called 1-7 days after their primary care visit for a musculoskeletal problem. All patients were administered PROMIS scales and PASS. The sample was 59.8% female, 49% were PASS Yes, and 47.5% were spine related problems. The average age was 54(17). Patients reported average PF of 44(8.1), PI of 59.2 (7.7), SE of 46.5(7.5) and SEAD 46(6.7). From receiver operator curves(ROC) the area under the curve(AUC) were calculated to indicate accuracy of predicting PASS. Subsequently, using thresholds (95%, 90%, 80% sensitivity/specificity) from the ROC analysis PROMIS scales were converted to binary variables to enter into a logistic regression to determine if a clinical decision rule for predicting PASS was useful (accuracy of determining PASS 70% or higher).

**Results**

The AUC for PROMIS domains were between 0.73-0.78 [PF=0.77(0.05), PI=0.78(0.05), SE=0.75(0.05), SEADL=0.73 (0.05)] suggesting greater than reasonable accuracy. All univariate correlations among PF, PI, SE, and SEAD were significant (r values ranged from 0.59-0.73). The best logistic regression models consistently retained PF and SE (accuracy for classifying PASS=70.3%) or PI and SE (accuracy for classifying PASS=72.3%) as independent predictors of PASS. Age and gender were not significant predictors.

**Conclusions**

A clinical decision rule using thresholds for PROMIS PI and SE scales is able to improve prediction of PASS for widely varying patients with musculoskeletal problems attending primary care with an accuracy of 72.3%. This data affirms that PASS status is influenced independently by self-efficacy, suggesting patient confidence in their ability to manage symptoms is equally as important as physical function and pain for patient recovery. Background:

**Keywords**

PROMIS, Physical Function, Pain Interference, Self-Efficacy, Patient Acceptable Symptom State

## P035 MCID Values of the PROMIS^®^ PF and PI in Orthopaedics

### Man Hung, Maren Voss, Shirley Hon, Jerry Bounsanga, Judith Baumhauer, Darrel Brodke, Richard Kendall, Nikolas Katzmers, Andrew Tyser, Charles Saltzman

#### University of Utah Department of Orthopaedic Surgery Operations, USA

**Background**

The minimal clinically important difference (MCID) is an important element of patient-reported outcome interpretation and it refers to the level of change that is considered meaningful from a patient or provider perspective. As yet there is little agreement on the best method for determining MCID. There is some evidence that condition type or disease severity does not greatly influence MCID levels. We applied comprehensive approaches to MCID estimation at multiple follow-up periods for sub-specialties of orthopaedics in foot, hand, and spine to determine various MCID values for the Patient-Reported Outcomes Measurement Information System (PROMIS) Physical Function (PF) and PROMIS Pain Interference (PI).

**Methods**

Consecutive patients aged 18 and older visiting a university orthopaedic center completed the PROMIS PF and PROMIS PI at first clinic visit and at follow-up visits. We estimated MCIDs using two distribution-based methods and two anchor-based methods at four follow-up periods. Patients were grouped based on level of change as indicated by a global rating of change measure used as the anchor question.

**Results**

The majority of MCID values from the different methods and follow-up points for the PROMIS PF ranged from 2-16 points in hand patients, 3-25 points in foot patients, and 3-20 points in spine patients. For majority of the PROMIS PI MCIDs ranged from 2-17 points for hand, 3-21 points for foot, and 1-21 for spine.

**Conclusions**

The smallest MCID values for each measure and specialty were obtained using 1/3 SD and ROC methods, and were all in the 2-3 point range regardless of orthopaedic specialty or follow-up time. The upper end of the MCID range showed more variability by specialty and patient type. MCID values at a mid-level of precision or lower are not likely to be impacted greatly by orthopaedic specialty, allowing similar MCID values to be applied across orthopaedic practice.

**Keywords**

Minimum Clinically Important Difference (MCID), Patient-Reported Outcome (PRO), PROMIS, Orthopaedic, Physical Function, Pain

## O036 Crosswalks of the PROMIS PF and Legacy Scales Across Orthopaedic Sub-Specialties

### Man Hung, Shirley Hon, Maren Voss, Jerry Bounsanga, Judith Baumhauer, Darrel Brodke, Richard Kendall, Andrew Tyser, Nikolas Katzmers, Charles Saltzman

#### University of Utah, Department of Orthopaedic Surgery Operations, USA

**Objective**

There are a number of joint or condition specific patient-reported outcome (PRO) measures used in orthopaedics. These PRO measures may be preferred for their ability to offer a brief and targeted assessment of the clinical area of interest. With the development of the Patient-Reported Outcomes Measurement Information System (PROMIS) Physical Function (PF) instrument and Computerized Adaptive Test (CAT) administration, it is possible to have a single instrument that can address the general needs of sub-specialties without a loss of precision and without added patient burden. The purpose of this study was to provide a crosswalk between the PROMIS PF and other commonly used PRO metrics in orthopaedics.

**Methods**

PRO instruments were delivered electronically to consecutive patients seeking care at an academic orthopaedic center between 2014 and 2017 at time of each visit as part of standard patient care. Linking was performed using graded-response IRT model and was used to transform the sub-specialty specific instrument scores into the PROMIS PF metric and to provide score conversion between these instruments.

**Results**

The PROMIS PF was correlated with the Foot and Ankle Ability Measure (FAAM) Activities of Daily Living (AD) subscale, the quick version of the Disabilities of the Hand and Shoulder (qDASH), and the Oswestry Disability Index (ODI).The measures were sufficiently uni-dimensional for IRT co-calibration. Crosswalk tables and mapping had been constructed to display the score linkage.

**Conclusions**

The development of crosswalks across new and previously used instruments encourages standardization of measurement, allowing the use of PROMIS CAT administration in future testing without a loss of data from previous patient testing. These crosswalks allow clinicians and patients, as well as researchers and administrators, the ability to interpret and understand the relationships between test scores and the ability to compare results from different studies.

**Keywords**

Crosswalk, Score Comparisons, Physical Function, PROMIS, FAAM, Qdash, ODI

## P037 Do PROMIS Physical Function Items Scale to Discriminate Level of Difficulty for Specific Mobility Tasks?

### Ryan Jacobson^1^, Michael Bass^3^, Judith F. Baumhauer^2^, Jeff Houck^1^

#### ^1^George Fox University, Newberg, OR, USA; ^2^University of Rochester, Rochester, NY, USA; ^3^Northwestern University, Chicago, IL, USA

**Background**

To evaluate the ability of specific Patient-Reported Outcomes Measurement Information System (PROMIS) Physical Function (PF) items to discriminate levels of difficuly for four fundamental mobility tasks—sit-to-stand, bending, walking and stair climbing.

**Methods**

PROMIS PF 2.0 item model parameters were obtained. A subset of 33 items aligned with one of four International Classification of Functioning (ICF) mobility codes—Sitting (d4103), Bending (d4105), Walking (d450) or Climbing (d4551). Three items were selected for each code as best corresponding with one fundamental mobility task, while also scaling in increasing difficulty across a maximized range of T-scores. Selection was iterative, aligning model parameters for potential item triads with T-score ranges. A stacked column chart was generated to visualize scaling of item triads for clinical interpretation.

**Results**

Stair climbing scaled best, discriminating increasing difficulty ratings across three selected items for a T-score range of 35-53. The most difficult stair item had a “No Difficulty” rating threshold at a T-score of 61.5. Walking discriminated for a range of 35-48 (“No Difficulty” threshold 52.8). Bending discriminated for ranges 27-33 and 38-49 (“No Difficulty” threshold 57.7). For *sit-to-stand*, PF items were unable to discriminate increasing difficulty. Broadening ICF coding to Changing Body Position (d410) allowed selection of three PF *transferring* items (bed-to-chair, stand from armless chair, squat then stand) that discriminated well for a T-score range of 22-42 (“No Difficulty” threshold 49.8).

**Conclusions**

Four item triads from the PROMIS PF item bank best discriminated difficulty in four highly relevant fundamental mobility tasks. Available PROMIS PF sit-to-stand items scaled poorly, limiting linkage between T-score and patient improvement in this important mobility task. “No Difficulty” thresholds fell below the average US population T-score of 50 only for *transferring*. Clinicians may use scaling of these item triads to make direct connections between improvements in patient T-score and physical abilities.

**Keywords**

PROMIS, T-Score, Physical Function, Physical Therapy, Mobility

## P038 Are PROMIS Scales Useful for Determining Success After Collaborative Physical Therapy/Primary Care Treatment?

### Ryan Jacobson, Dan Kang, Tyler Cuddeford, Jeff Houck

#### George Fox University, Newberg, OR, USA

**Background**

To assess the ability of PROMIS health domains [physical function (PF), pain interference (PI), self-efficacy of symptom management (SESM) and daily activities (SEDA)] to determine if patients with musculoskeletal (MS) problems consider their treatment a success after a PT/MD primary care intervention.

**Methods**

From March 2017 to September 2017, 88 patients were called 45-60 days after their primary care visit for a MS problem. A total of 63% were female, 41.3% had spine problems, and body mass index averaged 31.0(7.2) kg/m^2^. All patients were administered PROMIS scales and a Success question. The validated Success question asked patients to judge their outcome as “Not Helped” [NH] (n=23), “Improved” [Imp] (n=42), or “Partly Cured” or “Cured” [PCoC] (n=27). One way ANOVA models tested the association of PROMIS scores with Success responses (NH, Imp, PCoC). Receiver operator curves (ROC) were used to calculate the area under the curve (AUC) for determining patients that were “Not Helped”. Thresholds using 80% specificity were determined for patients “Not Helped”.

**Results**

PROMIS PI(p<0.01), SESM(p<0.01), and SEDA(p=0.017) showed significant differences between Success responses for each category. However, PF was not significant (p=0.08). Both PROMIS PI(11.7, p<0.01) and SE(7.7, p<0.01) showed the largest differences between patients considered NH and PCoC with less distinct differences between NH and Imp categories for PI(6.5, P<0.01) and SESM(4.6, p=0.02). The AUC for PROMIS PF, PI, SESM, and SEDA were the highest for identifying patients NH, ranging from 0.66-0.76. The PROMIS thresholds were PF 41.0, PI 61.0, SESM 42.0 and SEDA 42.7.

**Conclusions**

PROMIS scales were successful at discriminating patients’ judgement of Success after primary care treatment for musculoskeletal problems. The key predictors of patients “Not Helped” were PROMIS PI and SESM. The thresholds suggest benchmarks clinicians may use to judge when patients are likely not responsive to primary care MS treatment.

**Keywords**

PROMIS, Primary Care, Physical Therapy, Musculoskeletal, Multidimensional Assessment

## O039 The Impact of Lumbar Discectomy on Patient-Reported Outcomes: A Matched Cohort Study

### David S. Jevotovsky, Caroline P. Thirukumaran, Paul T. Rubery

#### University of Rochester Medical Center, Rochester NY, USA

**Objective**

When allocating a fixed amount of healthcare dollars, identifying the improvement surgery would provide is critical. Back pain patients are often thought to benefit less from surgery than other musculoskeletal patients as that they are more depressed, have less function and more pain. To evaluate the impact of lumbar discectomy (DSC) on patient-reported mood, function, and pain scores by comparing them to a matched cohort of patients undergoing arthroscopic anterior cruciate ligament reconstruction (ACLR).

**Methods**

Patients who underwent DSC or ACLR were retrospectively identified. PROMIS domains (PF, PI, Dep), patient demographics, and other encounter details were extracted. Primary outcomes (i) pre-operative PROMIS domain scores and (ii) scores at a minimum of 40 days post-operatively for DSC patients and 133 days post-operatively for ACLR patients, and (iii) the change in scores with surgery. Propensity score matching identified age-, sex-, race-, and comorbidity-matched groups from each cohort. Chi-square tests and non-parametric Kruskal-Wallis tests compared the distribution of outcomes and characteristics. Multivariate linear regression models with interactions between the matched cohort and operative phase estimated the change in the outcomes scores between the two cohorts.

**Results**

144 patients at a single academic medical center who underwent lumbar discectomy (n=88) or ACL reconstruction surgery (n=56) from February 2015 to July 2017 were identified. Age, gender, race, and Elixhauser co-morbidity index were similar between the matched cohorts (p>0.05). As compared to the ACLR cohort, the DSC cohort had lower adjusted post-operative PROM-PF scores (43.34 vs. 48.32) and higher adjusted post-operative PROMIS-PI (55.38 vs. 48.32) and PROMIS-D scores (46.2 vs. 39.2), indicating inferior outcomes. However, with respect to pre-operative scores, DSC patients experienced significantly greater improvement in PROMIS-PF (Adjusted estimate of interaction term: 3.35, 95% CI: 0.13 to 6.57, p=0.042). DSC patients experienced greater decline in PROMIS-PI (Adjusted estimate: --5.90, 95%CI: -9.14 to -2.66, p<0.001) and PROMIS-D scores (Adjusted estimate: -4.16, 95% CI: -7.60 to -0.72, p=0.018) with surgery.

**Conclusions**

DSC patients receive a larger benefit from surgery despite worse post-operative PROMIS scores and greater improvement than ACLR patients.

**Keywords**

PROMIS; Discectomy; Outcomes; Healthcare Value

## P040 A Simulation Study of DIF Detection Procedures

### Aaron J Kaat, Benjamin D Schalet

#### Northwestern University Department of Medical Social Sciences, USA

**Background**

The PROMIS standard for differential item functioning (DIF) has been hybrid logistic ordinal regression (i.e. lordif). However, several PROMIS banks have used other DIF procedures. It is common that different methods flag different items, without knowing which is correct. A simulation study allows direct evaluation of these procedures.

**Methods**

DIF was simulated under 14 different conditions in two hypothetical populations (28 simulations) for a 20-item fixed length test. DIF was simulated on 20% of items, varying type of DIF and focal population distribution differences with similar DIF-magnitude as previous studies (e.g. Woods, 2009). Items were flagged using five different methods. Errors (both over- and under-identification) were tracked, and generating theta score recovery was evaluated to gauge the impact of DIF on an individual’s score.

**Results**

The Wald and lordif chi-square tests were most likely to correctly identify DIF items, but also over-identified non-DIF items. For the other methods, non-uniform DIF was rarely detected. Both lordif pseudo-R2 (i.e. the PROMIS standard method) and the weighted area between the curve (wABC; both potential “impact” as opposed to detection indices) also failed to detect some cases of uniform DIF. Examination of theta recovery suggested that using group-specific parameters only improved when there was also a difference in focal population distributions. Indeed, ignoring DIF items (both uniform and non-uniform) but modeling group-specific distributions provided adequate theta recovery in 86% of the simulations.

**Conclusions**

DIF detection procedures often produce different results. The PROMIS standard appears to under-identify non-uniform DIF, but so do many of the other procedures. Surprisingly, the impact of DIF on an individual’s score was small even if item-level DIF was not included in scoring, provided that population differences were adequately accounted for. This has implications for PROMIS, where international researchers may detect language-based DIF and population differences from the US-based calibrations.

**Keywords**

Differential Item Functioning; Item Response Theory; PROMIS

## O041 Improving Detection of Individual Change with CATs: Score Precision Benefits and Response Burden Costs

### Michael A. Kallen, Karon F. Cook, Richard C. Gershon

#### Northwestern University Feinberg School of Medicine, USA

**Objective**

PROMIS CATs typically stop administering items after attaining a criterion score-precision level, currently set at standard error (SE) <3 (T-score metric). This precision level provides scores with an approximate reliability of 0.91, appropriate for group-level score comparisons and single-time-point observations. Accommodating increasing interest in using PROMIS CATs to detect individual-level score change across time may require producing more precise scores. We investigated score precision benefits and response burden costs associated with increasing CAT score reliabilities from 0.91 to 0.95.

**Methods**

We used current item parameters from two banks (PROMIS Physical Function (PF)-165 items; PROMIS Depression (DEP)-28 items) to simulate CAT administrations for N=1000 cases (standard normal distribution). For each bank, we compared mean and median # of items administered, mean score SE, and the correlation between CAT vs. full bank scores, using maximum allowed CAT score SEs of 2.99 vs. 2.24 (T-score metric), reflecting score reliabilities of 0.91 and 0.95, respectively.

**Results**

For PF, when increasing score reliability from 0.91 to 0.95, mean # of items administered increased (5.31 to 6.80), median # of items increased (4.00 to 5.00), the CAT vs. full bank score correlation increased (0.976 to 0.982) and mean score SE decreased (2.48 to 2.23, T-score metric). For DEP, mean # of items administered increased (6.01 to 7.81), median # of items increased (4.00 to 7.00), the CAT vs. full bank score correlation increased (0.977 to 0.985) and mean score SE decreased (2.91 to 2.64).

**Conclusions**

Although statistically significant score differences may not be clinically meaningful, patient- or clinician-based meaningful score differences may not be implementable if scores are insufficiently precise. Modest increases in average CAT length (PF: +1.5 items; DEP: +1.8 items) contributed to improved score reliability and an improved ability to detect individual-level score change (95% T-score CIs: 0.91 reliability-± 5.88; 0.95 reliability-± 4.38).

## P042 A New PROMIS^®^ Physical Function Short Form for Use in Relapse and Progressive Multiple Sclerosis Types

### Paul Kamudoni^1^, Dagmar Amtmann^2^, Karon Cook ^3^, Amy Barett, MSPH, MA^4^, Bimpe Olayinka-Amao Phar MPH^4^, Ari Gnanasakthy, MBA, ^4^, Rod Middleton^5^, Jeff Rodgers^5^, Jana Raab^1^, Oliver Guenther^1^, Christian Henke^1^

#### ^1^Global Evidence & Value Development – R&D, Merck KgaA, Darmstadt, Germany; ^2^Department of Rehabilitation Medicine, University of Washington, Seattle, WA, USA; ^3^Feinberg School of Medicine, Northwestern University, Chicago, IL, USA; ^4^Patient-Centered Outcomes Assessment, RTI Health Solutions, Durham, NC; ^5^UK MS Register, Swansea Medical School, Swansea, UK

**Objective**

The aim of this research was to develop and validate a short form based on the PROMIS physical function (PF) item bank, for use in MS.

**Methods**

This research followed a mixed method approach and involved multiple stages.

Step 1. Semi-structured concept elicitation (CE) interviews were carried out with patient with relapse-MS (n = 14 relapse-MS) from the US. Step 2. Concepts identified from the CE interviews were mapped to the PROMIS PF item bank to generate an initial pool of MS-relevant items. Subsequently, a panel of neurologists (n =6) rated the relevance of the shortlisted items, in MS. Then, a panel of measurement experts, assimilated results from the CE interviews, the rankings from and the neurologist panel, with prior information about the PF item bank (i.e., item information functions), to optimize coverage of the PF continuum by the item pool. Step 3. Cognitive debriefing (CD) interviews were carried out with MS patients (n = 24 relapse MS, n =24 primary progressive MS) from the US. Step 4. Two observational studies [cross-sectional study at two neurology clinics in the US, n = 300; and a longitudinal study based on the UK MS Register, n = 600] are being carried out to evaluate psychometric properties of the new short form.

**Results**

Eleven sub-domains relating to physical function (activities of daily living, upper-extremity, lower extremity functioning) were identified from the CE interviews [mean age = 44.1 years]. Initially, 48 items from the PROMIS physical function item bank matching concepts from the patient interviews were identified. Ratings by the neurologist expert panel (n =6) designated 38 items as the most relevant. Subsequently, the measurement experts resolved content overlaps and optimized the draft measure for targeting and reliability across levels of physical function, resulting in a total of 26 items. CD interviews confirmed the comprehensibility and comprehension of the short form.

**Conclusions**

This research has demonstrated that the PF item bank comprehensively covers all concepts considered relevant for relapse and progressive MS. Moreover, the current approach took advantage of prior empirical evidence related to the item bank, which further facilitated optimal targeting of the new short form.

**Keywords**

PROMIS; Physical function; item response theory; multiple sclerosis; patient-reported outcomes

## P043 Responsiveness of PROMIS^®^ Global Health Short Form (PROMIS10) in Systemic Lupus Erythematosus (SLE)

### Shanthini Kasturi^1^, Jackie Szymonifka^2^, Jessica R. Berman ^2, 3^, Kyriakos A. Kirou ^2, 3^, Alana B. Levine ^3^, Lisa R. Sammaritano ^2, 3^, and Lisa A. Mandl ^2, 3^

#### ^1^Tufts Medical Center and Tufts University School of Medicine, Boston, MA, USA; ^2^Hospital for Special Surgery, New York, NY, USA; ^3^Weill Cornell Medicine, New York, NY, USA

**Background**

The accurate and efficient serial measurement of patient-centered outcomes is a priority in the clinical care of SLE. We aimed to evaluate the responsiveness of PROMIS10, a 10-item universal patient-reported outcome measure of global physical and mental health, in SLE outpatients using patient and physician-derived anchors.

**Methods**

Adult SLE patients were recruited from an SLE Center of Excellence. Subjects completed PROMIS10 at two visits a minimum of one month apart. SLE disease activity was measured with a patient global assessment of change, a physician global assessment and the physician-derived SELENA-SLEDAI. Responsiveness over time of PROMIS10 scores was evaluated using known-groups validity. Effect sizes of changes in PROMIS global physical health and global mental health scores from baseline to follow up were compared across groups of patients who differed in their patient global assessment of change, physician global assessment, and SELENA-SLEDAI using Kruskal-Wallis tests.

**Results**

A diverse cohort of 223 SLE patients completed baseline surveys, with 186 (83%) completing a follow up survey. Using the patient-based anchor, PROMIS10 demonstrated mild to moderate responsiveness to improvement and worsening of health status for both global physical health (effect size 0.29, 0.0, and -0.27; p<0.001 for “better,” “same,” and “worse” health status respectively) and global mental health (effect size 0.29, 0.0, and -0.54; p<0.001). Using the physician-derived physician global assessment and SELENA-SLEDAI as anchors, there were no statistically significant differences in effect sizes across groups.

**Conclusions**

PROMIS10 showed responsiveness over time to patient-reported, but not physician-derived changes in lupus health status. These data suggest that PROMIS10 can be used to efficiently measure and monitor important aspects of the patient experience of lupus not captured by physician-derived metrics. Further studies are needed to evaluate the role of PROMIS in optimizing longitudinal disease management in SLE.

**Keywords**

Systemic Lupus Erythematosus, PROMIS, Patient-Reported Outcome Measures, Psychometrics

## O044 PROMIS^®^ Scales Detect Clinically Important Change Equal or Better as Compared to Disease Specific Patient Reported Outcomes in Patients after Knee Arthroscopy

### Raymond Kenney^1^, Brian Giordano^1^, Jeff Houck, PT^2^, Allison W. McIntyre, MPH^1^, Michael Maloney^1^

#### ^1^ University of Rochester Medical Center, USA; ^2^ George Fox University, USA

**Background**

Compare clinically important differences (CID) for PROMIS instruments (Physical Function(PF) and Pain Interference(PI)), and the Knee injury and Osteoarthritis Outcome Score (KOOS) using the established International Knee Documentation Committee (IKDC) scale as an anchor for change in subjects undergoing knee arthroscopy.

**Methods**

Patients undergoing knee arthroscopy at an ambulatory orthopaedic clinic were invited to participate. Subjects completed the PROMIS PF and PI instruments, KOOS and IKDC scales pre-operatively and post-operatively. Known minimal (11.5) and moderate (20.5) CID for IKDC improvement were used as anchor values. Receiver operator curve (ROC) analysis was applied to PROMIS and KOOS. Area under the curve (AUC) and thresholds that optimize sensitivity/specificity were used to compare PRO scales.

**Results**

88 subjects having surgery for meniscus tears, synovial plica, chondromalacia, or a loose body were enrolled. Average age 48.6 (11.7), BMI 30.9 (6.7) and 55.4% male. The change from pre-operative to last available follow up (2 weeks to 12 months) provided a range of responses and expected improvement.

The AUC values for minimal CID for PROMIS PF and PI were 0.88(0.04) and 0.85(0.04), respectively. This yielded minimal CID of 3.3 (PF) and 3.2 (PI). The AUC values for moderate CID in PROMIS PF was 0.86(0.04) and PI 0.89(0.04). This resulted in moderate CID of 5.2(PF) and -5.8(PI).

The KOOS subscales AUC for minimal CID was 0.76 to 0.90. The KOOS subscales minimal CID ranged from 12.5 to 17.5. The moderate KOOS subscales AUC varied from 0.76 to 0.89. The KOOS subscales moderate CID ranged from 14.3 to 22.5.

**Conclusions**

The accuracy of the PROMIS and KOOS scales were comparable. Additionally, the CID for both minimal and moderate CID were comparable to other studies. This suggests that PROMIS scales are able to detect change in knee arthroscopy similarly or better as compared to well-established disease specific scales.

**Keywords**

PROMIS, KOOS, Knee Arthroscopy

## P045 PAIN-CONTRoLS: A Patient-Informed Cryptogenic Polyneuropathy Clinical Trial using PROMIS^®^

### Kim S. Kimminau^1^ Mamatha Pasnoor^1^, Byron Gajewski^1^ , Lexie Brown^1^ , Laura Herbelin^1^ Omar Jawdat^1^, Pam Shlemon^2^ Mazen Dimachkie^1^, Richard J. Barohn^1^ and the PAIN-CONTRoLs Study Team

#### ^1^University of Kansas Medical Center, USA; ^2^Foundation for Peripheral Neuropathy, USA

**Objective**

Cryptogenic sensory polyneuropathy (CSPN) is a common, progressive neuropathy presenting with significant pain. The study’s objective was to determine which of four commonly prescribed medications is most effective and best tolerated. The study included continuous patient engagement to ensure meaningful outcomes were included in the design and analysis of findings.

**Methods**

We performed a prospective randomized open label comparative effectiveness study using a Bayesian adaptive design that included response adaptive randomization. At each interim analysis, a decision was made to either continue enrollment or stop the trial for success at baseline, weeks 4, 8 and 12. The primary outcome was a utility function which was a composite of efficacy and quits. 402 CSPN patients were randomized to nortriptyline (n=134), duloxetine (n=126), pregabalin (n=73), and mexiletine (n=69). Patients were asked during a focus group and via survey to identify patient-reported outcomes that should be used. Patients agreed with the investigator-selected choice of the PROMIS pain interference measure and suggested two additional PROMIS measures – fatigue and sleep interference.

**Results**

The utility functions were: nortriptyline 0.81 (95% credible interval 0.69-0.93, efficacy rates 25.4%, quit rates 38.1%); duloxetine 0.80 (95% credible interval is 0.68-0.92, efficacy rates 23.0%, quit rates was 37.3%); pregabalin 0.69 (95% credible interval 0.55-0.84, efficacy rates 15.1%, quit rates 42.5%), and mexiletine 0.58 (95% credible interval of 0.42- 0.75, efficacy rates 20.3%, quit rates 58.0%). Patients reported the primary reason for discontinuing participation was side effects including dry mouth, nausea, insomnia and fatigue.

**Conclusion**

If patients could stay on medication for three months, mexiletine had the best improvement in pain and fatigue. While there was no clear winner when efficacy and quits are combined, overall nortriptyline and duloxetine outperformed pregabalin and mexiletine. Patient collaborators provided effective input on patient-reported outcomes that resulted in capturing side effects using the PROMIS measures.

**Keywords**

Neuropathy, Patient Engagement, Side Effects, Bayesian Adaptive Design.

## O046 Does PROMIS^®^ Reflect Vocal Health Enough to Supplant Two Voice-Specific Quality of Life Instruments?

### Elliana Kirsh^1^, Thomas L. Carroll^1^, Chris Gibbons^2^, Jennifer J. Shin^1^

#### ^1^Department of Otolaryngology, Harvard Medical School, Boston, Massachusetts, USA; ^2^Department of Surgery, Harvard Medical School, Boston, Massachusetts, USA

**Objective**

To evaluate disease-specific (VHI-10, SVHI-10) and general (PROMIS) health status in patients reporting voice dysfunction, and determine whether PROMIS data alone can accurately represent vocal health in this population.

**Methods**

Adults (n=734) presenting to a tertiary care academic medical center laryngology subspecialty clinic completed the Voice Handicap Index-10 (VHI-10) and the Patient-Reported Outcomes Measurement Information System, PROMIS) 10-item general health instrument. Patients reporting concerns about their singing voice also completed the Singing Voice Handicap Index-10 (SVHI-10) survey. Patient characteristics and distributions of instrument scores were determined. The Spearman rho statistic was calculated to test the null hypothesis that there were no correlations between the VHI-10 or SVHI-10 and PROMIS scores. The potential for crosswalks was also assessed.

**Results**

The mean VHI-10 and SVHI-10 scores were 12.9 (SD 10.7) and 24.9 (SD 8.6), respectively. Mean PROMIS T-scores were 48.7 (SD 9.5) for physical health and 51.2 (SD 9.8) for mental health. PROMIS scores were 3.4 (SD 1.0) for global health, and 3.7 (SD 1.2) for the social item. VHI-10 scores were significantly correlated with all PROMIS component scores; social item scores were moderately correlated, with a Spearman rho of 0.37 (p<0.0001), while physical health (Spearman rho 0.29, p<0.0001), mental health (Spearman rho 0.28, p<0.0001), and global item (Spearman rho 0.20, p<0.0001) scores were also correlated. The VHI-10 item “I feel left out of conversations because of my voice” demonstrated moderate correlation with physical health (Spearman rho 0.30, p<0.0001), mental health (Spearman rho 0.32, p<0.0001), and the social item (Spearman rho 0.40, p<0.0001). There was no significant correlation between the SVHI-10 and PROMIS 10-item scores.

**Conclusions:** VHI-10 and PROMIS scores have weak to moderate correlations, and voice-related health may be related to multiple dimensions of general health. The PROMIS 10-item instrument may reflect the health status of patients with voice disorders, with limitations when singing-health is considered.

**Keywords:** voice, singing, validated instrument, otolaryngology, health status, quality of life

## O047 **Glenohumeral Osteoarthritis: The Utility of PROMIS**^**®**^**and the Influence of Mental Health**

### Eitan M. Kohan, J. Ryan Hill, Maria Schwabe, Alexander W. Aleem, Jay D. Keener, Aaron M. Chamberlain

#### Washington University in Saint Louis, USA

**Background**

The Patient-Reported Outcomes Measurement Information Systems (PROMIS) assessment includes computer adaptive tests that assess musculoskeletal function, pain interference, depression, and anxiety. The influence of mental health on patients’ self-reported pain and function has not been explored using PROMIS in patients with symptomatic glenohumeral osteoarthritis.

**Methods**

This cross-sectional study included 284 shoulders in 276 patients presenting with isolated glenohumeral osteoarthritis at a tertiary center. All patients completed the American Shoulder and Elbow Surgeons (ASES) score, Simple Shoulder Test (SST), Visual Analog Pain Scale (VAS), and PROMIS computer adaptive tests (CAT) at the time of presentation. PROMIS Anxiety and Depression scores were converted into GAD-7 and PHQ-9 scores, respectively, using the PROsetta stone crosswalk. Mean pain and functional scores were compared between patients with and without PROMIS-converted scores corresponding to a diagnosis of anxiety or depression, as well as between scores corresponding to varying severity of anxiety or depression.

**Results**

Compared to patients whose anxiety and depression scores were in the normal range, patients with PROMIS-converted scores corresponding to a diagnosis of anxiety or depression reported lower ASES, SST, Physical Function CAT (PFCAT), and Upper Extremity CAT (UECAT) scores and higher VAS and Pain Interference (PICAT) scores (*p*<.001). ANOVA analysis demonstrated lower overall ASES, functional ASES, SST, PFCAT, and UECAT scores as anxiety severity increased (*p*<.001). Similar results were seen with ASES and UECAT as depression severity increased (*p*<.001). Functional ASES (*p*=.004), SST (*p*=.001), and PFCAT (*p*=.002) were statistically significantly lower in those with moderate-to-severe depression. PICAT scores significantly increased as both anxiety and depression severity increased (*p* <.001 and <.01, respectively).

**Conclusions**

PROMIS-reported anxiety and depression scores correlate with lower functional and higher pain scores in patients with glenohumeral osteoarthritis. Further investigation is necessary to examine the influence that mental health has on outcomes following operative intervention in this population.

**Keywords**

Glenohumeral Arthritis, Shoulder, Outcomes, PROMIS, Depression, Anxiety, Function, Mental Health

## P048 Can Patient Reported Outcomes Guide Therapy Needs in Foot and Ankle Patients?

### Vasalos, Kostantinos, DPT^2^, Jillian Santer, DPT^2^, Judy Baumhauer, ^2^, Jeff Houck, PT, ^1,2^

#### ^1^George Fox University, Newberg, OR, USA; ^2^University of Rochester, Rochester, NY, USA

**Objective**

The objective of this analysis is to document the prevalance of patient acceptable symptom state (PASS) and determine the health domains that discriminate PASS patients and predict PASS state at the initiation of rehabilitation for foot and ankle problems.

**Methods**

Patient reported outcomes measurement information system (PROMIS) computer adaptive tests for physical function (PF), pain interference (PI), depression (Dep) and PASS ratings were identified from a large database. Of 746 unique patients, 114 patients had ICD-10 codes specific to the foot and ankle. Average age was 51(±18) years and 54.4% were female. ANOVA was used to evaluate differences in PROMIS scales by PASS state (Yes/No). The area under receiver operator curve (AUC) was used to determine the predictive ability of each PROMIS scale to determine PASS. Thresholds for near 95% specificity were also calculated for a PASS Yes state for each PROMIS scale.

**Results**

The prevalance of PASS Yes patients was 13.2% (15/114). Pass Yes patients were significantly better by an average of 7.2 to 8.0 points across all PROMIS health domains compared to PASS No patients. ROC analysis suggested that Dep (AUC=0.73(0.07) p=0.005) was the highest predictor of PASS status followed by PI (AUC=0.70(0.08) p=0.012) and PF (AUC=0.69(0.07) p=0.18). The threshold PROMIS t-score values for determining PASS Yes with nearest 80% specificity were PF = 56.1, PI = 45.7, and Dep = 45.1.

**Conclusions**

A small but important subset of patients (13.2%) identify at their initial physical therapy consultation as at an acceptable level of activity and symptoms. The PROMIS thresholds suggest patients are identified by pain and physical function slightly lower than the US population (T-Score 50). Clinicians may adapt their care to reinforce these patients self efficacy, set goals appropriate to their PF and PI scores, and use this information to prevent unnecessary costly rehabilitatoin.

**Keywords**

PROMIS; Patient Acceptable Symptom State (PASS); Physical Therapy

## P049 Measurement Equivalence of the Neuro-QoL Stigma for Children with Chronic Conditions

### Jin-Shei Lai^1^, David Cella^1^, Amy S. Paller^2^, Cindy Nowinski^1^

#### ^1^Northwestern University Department of Medical Social Sciences, USA; ^2^Northwestern University Department of Dermatology, USA

**Objective**

Stigma, defined as perceptions of self and publically enacted negativity, prejudice and discrimination as a result of disease-related manifestations, is commonly experienced by children with chronic health conditions. Yet few studies compare stigma across conditions, partly due to a lack of valid measures. To fill this void, we used differential item functioning (DIF) to evaluate measurement equivalence of the Neuro-QoL Stigma for children with skin conditions, neurological conditions (epilepsy, muscular dystrophy [MD], neurofibromatosis type 1 associated neurofibroma plexform [pNF]), and cancer.

**Methods**

Data from 842 children ages 8-17 years were analyzed. 110 had a diagnosis of epilepsy, 140 pNF, 43 82 cancer and 467 skin conditions (328 had atopic dermatitis/AD), with mean age (yrs)=13.5, 12.6, 14.1, 12.7 and 12.5, respectively. All completed the 18-item Neuro-QoL stigma except children with cancer and skin conditions. Two items inappropriate to these two conditions were not administered. DIF was conducted using lotdif package in R (criterion: χ2 >0.01, R2 change < 0.02) on gender, age (8-12 vs. 13-17 years), and conditions (reference group: AD). DIF impacts (theta differences between “all items included” versus “DIF items removed”) were evaluated to determine the inclusion/exclusion of DIF items.

**Results**

No items showed gender and age DIF. Five DIFs from three items were identified on the following comparisons: 1) pNF and epilepsy vs. AD; 2) non-AD skin conditions and pNF vs. AD; and 3) cancer versus AD. All DIFs were uniform with minimum impact (< 0.1 theta).

**Conclusions**

The Neuro-QoL Stigma exhibited stable measurement properties across various chronic conditions. The measure has now been re-calibrated by including children with non-neurological conditions.

**Keywords**

Children, Stigma, Chronic Conditions, Neuro-QoL

## P050 Validation of the Pediatric Patient-Reported Outcome Information System (PROMIS^®^) Pain Interference, Mobility and Upper Extremity Item Banks in the General Dutch Population

### M.A.J. Luijten^1,2^, C.B. Terwee^2^, L. Haverman1, L.D. Roorda^3^, R.R.L. van Litsenburg^4,5,^ M.A. Grootenhuis^4^

#### ^1^Emma Children’s Hospital Amsterdam UMC, University of Amsterdam, Psychosocial Department, Amsterdam, The Netherlands; ^2^Amsterdam UMC, location VUmc, Department of Epidemiology and Biostatistics, Amsterdam, The Netherlands; ^3^Amsterdam Rehabilitation Research Center | Reade, Amsterdam, The Netherlands; ^4^Princess Maxima Centre for Pediatric Oncology, Utrecht, the Netherlands; ^5^ Amsterdam UMC, location VUmc, Department of Pediatric Hematology-Oncology, Amsterdam, the Netherlands

**Background**

Our aim was to validate the pediatric V2.0 PROMIS Pain Interference, Mobility and Upper Extremity item banks, in the general Dutch population.

**Methods**

Children 8-18 years old (n = 1326), divided into two age groups (8-12, 13-18) representative of the Dutch population on key demographics (age, sex, ethnicity and education level) were asked to complete the PROMIS Mobility, Upper Extremity and Pain Interference item banks (consisting of 24, 34 and 19 items respectively) and the Pediatric Quality of Life Inventory (PedsQL). The assumptions of unidimensionality (using CFA), local independence (residual correlations) and monotonicity (Mokken scale analysis) were assessed. DIF for gender was assessed. The item fit of the GRM model was assessed (S-X^2^, p-value > .001). High correlations (Pearson’s *r* >.70) were expected between the PROMIS T-scores and the PedsQL Physical subscale. Weaker correlations (Δ*r* >.10) were expected with other PedsQL subscales.

**Results**

The final sample (n=555) was representative of the Dutch population (within 2.5%). The Mobility and Upper Extremity data was skewed. Unidimensionality was met for all item banks. Local dependence was present in the Mobility and Upper Extremity item banks (28 and 14 pairs, respectively). The assumptions of monotonicity were met. No DIF was found for gender and there was no item misfit. The Mobility item bank correlated highly (*r*=0.71) and the Pain Interference and Upper Extremity had a moderately strong correlation (*r*=-0.53, *r*=0.51, respectively) with the PedsQL Physical subscale. Pain Interference also had a moderately strong correlation with the PedsQL Emotional subscale (r=-.47). All other correlations were substantially weaker (Δ*r* >.10).

**Conclusions**

The Dutch version of the pediatric Pain Interference, Mobility and Upper Extremity item banks displayed satisfactory psychometric properties In a Dutch normative sample. More data is required for estimating stable parameters, due to skewness. Higher difficulty items might be required for the Dutch population.

**Keywords**

PROMIS, Pain Interference, Mobility, Upper Extremity, pediatric

## P051 Validation of the Pediatric Patient-Reported Outcome Information System (PROMIS^®^) Peer Relationship Item Bank in the General Dutch population

### M.A.J. Luijten^1,2^, R.R.L. van Litsenburg^3,4^, C.B. Terwee^2^, M.A. Grootenhuis^3^, L. Haverman^1^

#### ^1^Emma Children’s Hospital Amsterdam UMC, University of Amsterdam, Psychosocial Department, Amsterdam, The Netherlands; ^2^Amsterdam UMC, location VUmc, Department of Epidemiology and Biostatistics, Amsterdam, The Netherlands; ^3^Princess Maxima Centre for Pediatric Oncology, Utrecht, the Netherlands; ^4^ Amsterdam UMC, location VUmc, Department of Pediatric Hematology-Oncology, Amsterdam, the Netherlands

**Background**

Our aim was to validate the pediatric V2.0 PROMIS Peer Relationship item bank in a general Dutch pediatric population.

**Methods**

Children 8-18 years old (n = 1324), divided into two age groups (8-12, 13-18) representative of the Dutch population on key demographics (age, sex, ethnicity, and education level), were asked to complete the PROMIS Peer Relationship item bank (15 items) and the Pediatric Quality of Life Inventory (PedsQL). The assumptions of unidimensionality (using CFA and bi-factor analysis), local independence (residual correlations) and monotonicity (Mokken scale analysis) were assessed. DIF was assessed for gender The item fit of the GRM model was assessed (S-X^2^, p-value > .001). For construct validity, high correlations (Pearson’s *r* >.70) were expected between the Peer Relationship T-score and PedsQL Social subscale. Lower correlations (Δ*r* >.10) were expected with the other subscales. Reliability of the full bank was calculated with the standard error of measurement (SEM) of theta.

**Results**

The final sample (n=527) was representative of the Dutch population (within 2.5% of population numbers). Unidimensionality was not conclusively met by CFA (CFI=.95, TLI=.94, RMSEA=.11), but was accepted after fitting a bi-factor model (omega H=0.88, ECV=0.81). The assumptions of monotonicity and local independence were met. No DIF was found for gender. One (reversed) item: “I played alone and kept to myself”, displayed item misfit (S-X^2^(80)=140.11, *p*< .001)A moderate (*r* = 0.59) correlation was found between the item bank and the PedsQL Social subscale. Correlations with other subscales were substantially lower (Δ*r* >.10). The SEM of the full-length item bank was satisfactory (< .32) for 87.6% of the patients. Ability estimates were most reliable in the direction of clinical interest.

**Conclusions**

The Dutch version of the pediatric Peer Relationship item bank displayed satisfactory psychometric properties in a Dutch normative sample. One item might require revision in terms of formatting.

**Keywords**

PROMIS, Peer Relationships, Pediatric, Validation, Dutch

## O052 Impact of CIDP: PROMIS^®^ Physical Function SF-4 and I-RODS Analysis from a US Patient Survey

### Rajiv Mallick^1*^, Ingemar Merkies^2,3^, Anne Haudrich^1^, Ann Bullinger^1^, Daniel Riley^4^, and Catharina Faber^2^

#### ^1^CSL Behring, King of Prussia, PA, USA; ^2^Maastricht University, School for Mental Health and Neuro Sciences (MHeNS), Maastricht, Netherlands; ^3^Sint Elisabeth Hospital, Willemstad, Curaçao; ^4^Bryter, London, UK

**Background**

Chronic inflammatory demyelinating polyneuropathy (CIDP), is a rare condition involving neuromuscular disability which affects physical function and activity/participation.

**Methods**

Survey data from 475 US adults with self-reported CIDP, recruited by the GBS/CIDP Foundation, was used to evaluate diagnosis timing, treatment, and impact on (a) physical function measured using the PROMIS Physical Function (PF) Short Form-4 and (b) daily activity/participation measured using the Inflammatory Rasch-built Overall Disability Scale (I-RODS), containing activities ranging from ‘easiest’ (*reading*) to most ‘difficult’ (*running*). Patients were characterized by observed tertiles of PROMIS PF T-scores and I‑RODS centile scores in terms of (a) time from initial symptoms to CIDP diagnosis and (b) impact on work/living conditions.

**Results**

Mean age at diagnosis: 51 years. Median time between first recognized symptoms and diagnosis: 7 months (>1 year for 39% of patients). PROMIS PF mean T-score was 37.0 (standard deviation [SD] = 8.4); tertiles: 23–33, 34–39, 40–57, compared with a US population norm of 50. I-RODS centile score was 57.4 (SD 17.5); tertiles: 6–47, 48–61, 63–100. Compared with the lower tertiles, patients in the ‘best’ tertile had a lower probability of: >12 vs. ≤12 months from initial symptoms to official diagnosis of CIDP (PROMIS: 30% vs. 43% [p=0.0064]; I-RODS: 33% vs. 42% [p=0.0527]); making changes to employment (both scales: 44% vs. 68% [p<0.0001]); needing alterations to their residence (PROMIS: 23% vs. 48%; I-RODS: 22% vs. 49% [Both scales: p<0.0001]; needing to move home due to their CIDP (PROMIS: 13% vs. 29% [p=0.0002]; I-RODS: 13% vs. 30% [p<0.0001]).

**Conclusions**

These findings demonstrate patients with CIDP had worse physical function than the general US population. Those among the best third for physical function or activity/participation had been diagnosed sooner, and made fewer changes to employment or living conditions on account of their CIDP.

**Funding**

CSL Behring sponsored the study

**Keywords**

Chronic Inflammatory Demyelinating Polyneuropathy, Inflammatory Rasch-built Overall Disability Scale, PROMIS Physical Function Short Form (SF-4), Disability, Delayed Diagnosis

## P053 Can Pre-Surgery PROMIS-29 Scores Identify Patients Likely to do Well after Total Joint Replacements?

### Lisa A. Mandl, MPH^1,2^, Charles N. Cornell^1,2^, Michael B. Cross^1,2^, Alejandro Gonzalez Della Valle^1,2^, Mark P. Figgie^1,2^, Seth A. Jerabek^1,2^, Justin T. Do, BSE^1^, Mayu Sasaki, MPH^1^, Nathaniel Hupert, MPH^2^, Jackie Szymonifka, MA^1^, Steven K. Magid^1,2^

#### ^1^Hospital for Special Surgery, New York, NY, USA; ^2^Weill Cornell Medicine, New York, NY, USA

**Background**

We would like to determine whether pre-operative PROMIS29 domains are associated with either serious adverse events or clinical outcomes 1-year after total hip or total knee replacement, (TKR and THR).

**Methods\**Community-dwelling patients ≥65yo scheduled for elective TKR or THR were recruited from a musculoskeletal specialty hospital. PROMIS29 and Hip/Knee Injury and Osteoarthritis Outcome Score (HOOS/KOOS) were administered pre-operatively and at 1-year. Adverse events were obtained from medical records and by phone. Regression models were created by considering all variables which were significant at the 0.05 level in univariate models, and then performing backward selection to retain variables with 0.05 significance. Age and sex were forced in to all models.

**Results**

740 subjects, 303 THR and 437 TKR enrolled. Mean age 72 years (range 65-94), 95.1% Caucasian, 63.5% female, and 9.0% had > 1 severe adverse event at 1 year. Controlling for age, gender, and which joint was replaced, pre-operative PROMIS29 pain intensity predicted being an OMERACT-OARSI responder at 1-year, (OR 1.6; 1.3-2.0). No PROMIS29 score predicted HOOS 1-year outcomes in THR. Among TKR, pre-operative PROMIS29 Fatigue was associated with 1-year KOOS Symptoms and Quality of Life scores, (p=0.02 and p<0.001), PROMIS29 Depression was associated with 1-Year KOOS Pain and Activities of Daily Living, (p=0.003 and p= 0.001). Pre-operative PROMIS29 Physical Function was also associated with KOOS 1-year ability to Perform Sport and Recreation, (p=0.002). In a multivariable regression, pre-operative PROMIS29 Depression scores were also significantly associated with 1-year SAE in THR cases, (OR 1.09; 1.02-1.17).

**Conclusions**

Multiple PROMIS29 domains predicted functional outcomes after TJR, including whether THR patients met OMERACT-OARSI responder criteria. Pre-operative PROMIS29 depression scores also predicted 1-year serious adverse events in THR. PROMIS29 may be an efficient tool to risk stratify this patient population in busy clinical practice.

**Keywords**

PROMIS29, Arthroplasty, Osteoarthritis, Pain, Function

## O054 PROMIS-29 as a Predictor of High Risk Hip Fracture Repair Patients

### Lisa A. Mandl, MPH^1,2^, Serena Lian, BS^2^, Kirsten Grueter, BSN^2^, Jackie Szymonifka, MA^2^, Joseph M. Lane^1,2^

#### ^1^Weill Cornell Medicine, USA; ^2^Hospital for Special Surgery, USA

**Objective**

To evaluate whether PROMIS-29 predicts short-term mental and physical health states after hip fracture surgery.

**Methods**

PROMIS-29 was administered to cognitively intact patients >65 years old, 2-4 days after surgery for low trauma hip fracture. Answers related to the week prior to fracture. Cumulative adverse events were measured through 30 days. PROMIS-29, Three-Item Loneliness Scale, Lubben Social Networks Scale (LSNS-18), and Falls Efficacy Scale (measures fear of falling) were administered at 3 months. Data analyzed using t-tests, Wilcoxon rank-sum tests, and Spearman correlations.

**Results**

203 patients, 71.9% female, 91.6% Caucasian, median age 81.8. At 3 months. 4.8% died and 24.3% had > 1 serious adverse event (SAE). Patients who died had worse baseline PROMIS-29 Physical Function (34.4 vs 45.3; p=0.007) and trended towards worse PROMIS-29 Fatigue (43.1 vs.58.8; p=0.07). Patients with SAE had worse baseline Physical Function (38.5 vs. 48.0; p<0.001), Pain Interference (49.6 vs. 41.6; p=0.006), and Pain Intensity (3 vs. 0; p=0.002). Baseline PROMIS-29 Anxiety and Depression were correlated with PROMIS-29 Anxiety and Depression at 3 months (r=0.37 and r=0.41; both p<0.001). Baseline Anxiety and Depression were both strongly and significantly correlated with worse subjective loneliness at 3 months (r=0.52 and r=0.59; both p<0.001), but were not correlated with social isolation. In addition, baseline Depression, Anxiety, and Physical Function were strongly correlated with fear of falling at 3 months (r=0.46, r=0.40, r=-0.56; all p<0.001).

**Conclusions**

Baseline PROMIS-29 scores were associated with mental and physical health status after surgery for an unexpected hip fracture. They were also associated with fear of falling at 3 months, a strong predictor of future falls. Interestingly, PROMIS-29 scores were also associated with loneliness but not social isolation; loneliness is associated with incident frailty and worsening physical function. PROMIS-29 is a parsimonious instrument to effectively identify at-risk patients in this vulnerable population.

**Keywords**

PROMIS29, Arthroplasty, Osteoarthritis, Pain, Function

## P055 Use of Google Analytics to Evaluate HealthMeasures.net as a PROMIS^®^ Dissemination Tool

### Christa Martens, MPH^1^, Nan Rothrock^1^, Kelly Johnston, MPH^2^

#### ^1^Northwestern University, USA; ^2^University of Pittsburgh, USA

**Background**

The objective of this work was to evaluate the successes of HealthMeasures.net as a digital platform to disseminate PROMIS^®^ and other HealthMeasures-specific information.

**Methods**

Google Analytics were used to analyze HealthMeasures.net user engagement statistics from March 1, 2016-March 31, 2018. Website pages was divided into four categories: application, descriptive, educational, and product. Across categories, the following metrics were analyzed: new and returning user engagement, number of sessions, average time spent onsite per session, number of pages visited per session, and average time per page. Sessions were used as the primary selection metric and the 50 pages with the most sessions were evaluated.

**Results**

Over 2 years, HealthMeasures.net visitors engaged in 360,999 sessions with 1,549,200 total page hits. Pages with the highest number of sessions include 25 descriptive, 12 applications, 9 product, and 4 educational pages. Descriptive pages, such as the PROMIS landing page, drew the highest number of sessions for new and returning visitors, averaging 10,821 sessions per destination page. Product pages, such as the Search and View Measures, averaged longer sessions and the most page visits per session, with new visitors who completed a search averaging 93 page visits per session. Visitors to application or educational pages spent an average of 30 seconds longer on a page than visitors to descriptive or product pages. Sessions that included application pages were dominated by returning visitors, with the exception of PROMIS scoring pages which both new and returning users heavily engaged in, averaging 2:03-3:33 minutes per page.

**Conclusions**

HealthMeasures.net visitors seek descriptive information about PROMIS and other HealthMeasures most frequently, but spend the most time on pages with information about applying PROMIS and other HealthMeasures to their work. Product pages are effective for engaging new visitors in the pursuit of more information.

**Keywords**

PROMIS, patient-reported outcomes, google analytics, website

## P056 PRO*gram* Management for Ongoing PRO Collections in a Clinical Setting

### Allison W. McIntyre, MPH, Judith Baumhauer, MPH

#### University of Rochester Medical Center, USA

**Background**

To maintain high quality clinical collection of Patient-Reported Outcomes (PRO) it is important to develop a framework to support the acquisition of data.

**Methods**

Collecting PRO from thousands of patients a month is a great accomplishment, however, maintaining collections, adding new collection sites and keeping providers and staff informed is, arguably, as important as your collection platform. With input from physicians and staff we identified key areas to support ongoing collection of PRO.

**Results**

PRO*gram Initiation* is where it begins as new sites are established. Integration between IT and the Program Manager is imperative to assure each site gets started with an effective and efficient collection scheme. PRO*gram Maintenance* is a hands-on approach to reinforce lessons learned at initiation. Support is provided for all sites at scheduled intervals after initiation and as needed thereafter. To provide a culture of continuous learning, we PRO*mote Education* for the providers, staff and patients. Understanding why the data is being collected and how it can be used results in invested participants. PRO*gram Monitoring* is one of the most important components. It allows the PRO team, as well as providers and departments, to track process measures, including administration and completion rates. This information is also available to the Project Manager to identify potential problems that can be addressed before they result larger issues. Finally, the robust dataset produced by this successful collection process provides an opportunity to PRO*be Data* for quality improvement and research.

**Conclusions**

Developing a system that allows us to monitor and support PRO collections across the institution allows the 774 unique providers, who have collected data, to review the 1.8 million PRO scores, collected to date, on 204,086 unique patients. Continuous PRO*gram management* maintains the momentum necessary for such a large enterprise to be successful at large-scale PRO collection and use.

**Keywords**

Patient-Reported Outcomes, Program Management

## O057 Precursors to Driving Cessation: Are They Associated with Poorer Health-related Quality of Life?

### Thelma Mielenz^1^, David Strogatz^2^, Dagmar Amtmann^3^, Lisa Molnar^4^, Lindsay Ryan^5^, David Eby^6^, Carolyn DiGuiseppi^7^, Marian Betz^8^, Linda Hill^9^, Guohua Li^10^

#### ^1^Columbia University, New York NY, USA; ^2^Bassett Healthcare Network, Cooperstown, NY, USA; ^3^University of Washington, Seattle, WA, USA; ^4^University of Michigan, Ann Arbor, MI, USA; ^5^University of Michigan, Ann Arbor, MI, USA; ^6^University of Michigan, Ann Arbor, MI, USA; ^7^University of Colorado, Aurora, CO, USA; ^8^University of Colorado, Aurora, CO, USA; ^9^University of California, San Diego, CA, USA; ^10^Columbia University, New York NY, USA

**Objective**

Research to address a potential association between negative driving outcomes and the broader concept of health-related quality of life (HRQOL) is needed. We hypothesize that individuals who report less driving space and more driving crashes will report a lower quality of life compared to those with positive driving outcomes.

**Methods**

This study uses baseline LongROAD data (prospective cohort with 2990 drivers aged 65-79 years). The outcome was the 8-domain PROMIS-29 Adult Profile. Multiple linear regression models calculated adjusted means for each of the PROMIS-29 outcomes by driving space and by crash status. We adjusted for demographics, vision, correct word recall, driving importance, days driven per week, miles driven per week and the standard errors by site.

**Results**

Participants with one or more crash had a higher adjusted mean or more pain for Pain Interference (47.8 (n=320)) compared to those with no self-report of crashes (46.3 (n=2460)*, P*=0.275)). Participants with one or more crash had a higher adjusted mean or more pain for Pain Intensity. (2.22 (n=320)) compared to those with no self-report of crashes (1.86 (n=2471)*, P*=0.0064)). Participants with less driving space had a lower adjusted mean or less physical function for Physical Function (49.9 (n=622)) compared to those with a more self-reported driving space (51.2 (n=2160)*, P*=0.0217)). Participants with less driving space also had a higher adjusted mean or more depressive symptoms for Depression (44.3 (n=626)) compared to those with a more self-reported driving space (43.6 (n=2173)*, P*=0.0367)).

**Conclusions**

More crashes and less driving space were associated with more pain, less physical function and more depressive symptoms. These differences in the adjusted means were significant but not necessarily clinically meaningful. Besides impacting mental health (depressive symptoms), negative driving outcomes may also impact physical health (pain and function) with PROMIS-29 broadening the concept of HRQOL in driving research.

**Keywords**

Driving Space, Crashes, Older Drivers, PROMIS-29

## P058 Patient Reported Outcomes, Item Response Theory, Model Assumptions, Latent Variable, Non-Normality Construct validity of the PROMIS^®^ scales for Taiwanese using CFA

### Ay-Woan Pan^1^, Tsyr-Jang Chen^2^

#### ^1^School of Occupational Therapy, College of Medicine, National Taiwan University, Taiwan; ^2^Department of Mechanical Engineering, LungHwa University of Science and Technology, Taiwan

**Background**

The purpose of this study is to evaluate the construct validity of five PROMIS measures in persons with and without mental disorders in Taiwan.

**Methods**

Three hundred and nine community sample, who did not report any mental illness conditions were recruited (mean age 27.8 ± 9.48 years). The subjects with mental disorders were recruited from mental health clinics and community based residential settings. Three hundred and twenty-two subjects with mental disorders (mean age 47 ± 11.5 years) were recruited from mental health clinics and community based residential settings (IRB approval, 201405051RINC). Thirty-five percent of the subjects were college graduates; Seventy-two percent of the subjects were single. The average self-rated quality of life score was 74. LISREL and SPSS were used for the subsequent analysis.

**Results**

The results showed that all scales presented excellent internal consistency with Cronbach’s alpha value over 0.9 in the total. All items had a strong correlation with their own scale except some had mildly low item-total correlation (e.g. 0.4). The RWSEA, NFI, CFI, GFI, and AGFI of depression, anxiety, anger, sleep disturbance, and sleep related impairment scales were 0.11, 1.00, 1.00, 0.99, 0.99; 0.11, 1.00, 1.00, 0.99, 0.99; 0.14, 1.00, 1.00, 0.97, 0.96; 0.16, 1.00, 1.00, 0.97, 0.97; 0.18, 1.00, 1.00, 0.96, 0.95. There were significant differences on scores of anger, sleep disturbance and sleep related impairment scales between two groups which the healthy subjects had worse condition than persons with mental illness on anger and sleep related impairment.

**Conclusions**

The findings of this study supported the construct validity of depression, anxiety, anger, sleep disturbance and sleep related impairment scales. Further implication of the differences between healthy subjects and persons with mental illness on anger and sleep related impairment will be mentioned.

**Keywords**

PROMIS, Construct Validity, CFA

## P059 PROMIS^®^ Pediatric and Parent Proxy Global Health Translations: Challenges and Solutions

### Emily Parks-Vernizzi^1^, Benjamin Arnold, MA^1^, Helena Correia^2^

#### ^**1**^FACITtrans, Elmhurst, IL, USA; ^2^Northwestern University Feinberg School of Medicine, Department of Medical Social Sciences, Evanston, IL, USA

**Objective**

The PROMIS^®^ Pediatric and Parent Proxy Global Health scales assess a child’s overall physical, mental, and social health. Multilingual translations of these measures will enable international pediatric studies and clinical use in a variety of countries. The objective of this presentation is to report on a sample of these translations, discuss issues arising from linguistic validation across multiple languages, and provide guidance for future translations.

**Methods**

These measures were translated into Afrikaans, Dutch, French, Italian, Japanese, Korean, Portuguese, Russian and Spanish, according to the FACIT translation methodology. Translations were tested with five native-speaking pediatric and five adult participants from the general population of each language. Participants completed the relevant scale and participated in a cognitive debriefing interview. Qualitative analyses of participant comments determined the linguistic equivalence of each translation and provided insight into the relevance of the concept in each language.

**Results**

Translated items were well understood by participants in each sample. Translations were revised for the pediatric measure as needed (and consistency maintained as necessary on the Proxy measure) if participants’ comments revealed misunderstanding of an item’s intended meaning. For example, the Japanese translation of “in general” was revised to “usually.” Some children commented that “health” and “physical health” were identical in meaning. The terms “rate,” “quality of life” and “mood” required alternative translation solutions to ensure proper register for children, cultural appropriateness, conceptual equivalence and harmonization across languages.

**Conclusions**

The translated versions of the PROMIS^®^ Pediatric and Parent Proxy Global Health measures are conceptually equivalent to the English source. Concurrent assessment of children’s and parent’s item interpretation confirmed consistent understanding between pediatric and proxy versions. The translated measures can be used in research, multinational trials, and clinical practice.

**Keywords**

Pediatric, Proxy, Global Health, Linguistic Validation, PROMIS

## P060 Differential Item Functioning by Language for PROMIS^®^ Physical Function Items: Application of a Two-Step Wald Approach

### John Devin Peipert^1^, Aaron Kaat^1^, Felix Fischer^2^, Sandra Nolte^2^, Caroline B. Terwee^3^, Ron D Hays^4^, Claude Messan Setodji^5^, Benjamin Schalet^1^

#### ^1^Department of Medical Social Sciences, Feinberg School of Medicine, Northwestern University, Chicago, IL, USA; ^2^Department of Psychosomatic Medicine, Charité - Universitätsmedizin Berlin, Berlin, Germany; ^3^Department of Epidemiology and Biostatistics, VU University Medical Center, Amsterdam, The Netherlands; ^4^University of California, Los Angeles, Los Angeles, CA, USA; ^5^RAND Corporation, Pittsburgh, PA, USA

**Background**

This study used a two-step Wald approach to identify differential functioning (DIF) between United States (US) English, German, and Dutch versions of PROMIS Physical Function items.

**Methods**

We examined DIF on PROMIS Physical Function items commonly used in short forms in US English (n=808), German (n=266), and Dutch (n=1995) using the FlexMIRT software. A two-step Wald approach was used to determine DIF in item parameters. The first step employs the Wald-2 approach to identify anchor items (items free of DIF). The Wald-2 fits two separate, multiple group graded response models, one with item parameters constrained to be equal across groups and one with parameters freed, then uses a Wald χ^2^ statistic to flag items for DIF; items with significant Wald χ^2^ at this stage are flagged as DIF items and the remainder are retained as anchor items. Then, the process is repeated comparing only items demonstrating DIF in step 1 with the Wald χ^2^.

**Results**

Most of the items in the analysis demonstrated DIF. In general, overall DIF was driven by uniform DIF (constant DIF across trait levels) versus non-uniform DIF (non-constant DIF across trait levels). For example, the Wald χ^2^ for US English vs. Dutch on item PFA11 (“Are you able to do chores such as vacuuming or yard work?”) slope parameter was χ^2^ = (1, N=2803) = 1.6, p=0.20. However, the Wald χ^2^ for this item’s intercept was χ^2^ = (4, N=2803) = 107.1, p<0.001.

**Conclusions**

Future implementations of the German and Dutch versions of some PROMIS Physical Function items should consider the potential for DIF in comparison to US English. These results will be compared to other DIF methods, including an approach using propensity score matched samples and quantifications of DIF magnitude using multiple approaches.

**Keywords**

Differential item functioning, item response theory, physical function

## O061 Exploring the Potential of A Mixture-Computerized Adaptive Test for Use in Heterogeneous Populations

### Richard Sawatzky^1,2^, Bruno D. Zumbo^3^, Tolulope Sajobi^4^, Lara Russell^2^, Anne Gadermann^2,3^ Jacek Kopec^3^, Juxin Liu^5^, Pamela Ratner^3^, Amery Wu^3^, Lisa M. Lix^6^

#### ^1^Trinity Western University, Canada; ^2^Centre for Health Evaluation and Outcome Sciences, Canada; ^3^University of British Columbia, Canada; ^4^University of Calgary, Canada; ^5^University of Saskatchewan, Canada; ^6^University of Manitoba, Canada

**Background**

Computerized adaptive tests (CATs) are increasingly used for measuring patient-reported outcomes (PROs) in heterogeneous populations. CAT-predicted PRO scores may be inaccurate when sources of heterogeneity (e.g., sex, age, health status) are ignored. The Draper-Lindley-de Finetti (DLD) framework of measurement validation provides a theoretical context for applying latent variable mixture models (LVMMs) for obtaining heterogeneity-adjusted CAT scores. Our objectives are to examine benefits and challenges of applying LVMMs to estimate heterogeneity-adjusted CAT scores and to compare them to unadjusted scores.

**Methods**

Data for our expository analysis were based on responses to 39 items of the daily activities domain of the CAT-5D-QOL. Respondents (N = 1,666) were recruited from a rheumatology clinic (20%), a waiting list for knee or hip replacement (20%), and a random stratified community sample (60%) in Canada. LVMMs were applied by specifying a mixture polytomous item response theory (IRT) model with difficulty and discrimination parameters free to vary across latent classes. The LVMM parameter estimates were used to program a “mixture-CAT” for obtaining scores that are adjusted for probability of class membership. Simulation was used to evaluate accuracy of scores.

**Results**

A 2-class LVMM resulted in improved model fit, relative to a 1-class model (class proportions were 0.64 and 0.36). Latent class membership was only partially predicted by several health status variables and age. Relative to a conventional CAT based on 1-class IRT parameter estimates (assuming no heterogeneity), a mixture-CAT based on the 2-class LVMM parameter estimates (accommodating population heterogeneity) resulted in improved accuracy.

**Conclusions**

Mixture-CATs could lead to improved accuracy of PRO scores in heterogeneous populations. Related benefits may include improved efficiency and diversity in item selection. However, these benefits rely on the ability to predict latent class membership, which could be a challenge. The results provide impetus for further research on mixture-CATs for measuring PROs.

**Keywords**

Computer adaptive test; latent variable mixture models; item response theory; population heterogeneity

## O062 Differential Item Functioning by Language for PROMIS^®^ Physical Function: Applying Ordinal Logistic Regression and Monte Carlo Simulations in Lordif

### Benjamin D. Schalet^1^, Aaron Kaat^1^, John Devin Peipert^1^, Caroline B. Terwee ^2^, Felix Fischer^3^, Sandra Nolte ^3^, David Cella^1^

#### ^1^Department of Medical Social Sciences, Feinberg School of Medicine, Chicago, USA; ^2^Department of Epidemiology and Biostatistics, VU University Medical Center, Amsterdam, The Netherlands; ^3^Department of Psychosomatic Medicine, Charité - Universitätsmedizin Berlin, Berlin, Germany

**Background**

The PROMIS standard for differential item functioning (DIF) is hybrid logistic ordinal regression (with lordif in R); DIF analysis validates score comparability across languages.

**Methods**

To detect and evaluate language DIF, we subjected data in US-English (n=808), German (n=266), and Dutch (n=1995) for 19 commonly used PROMIS Physical Function items to iterative ordinal logistic regression, item response theory estimation/scoring, and Monte Carlo simulations in lordif. We first applied the conventional threshold of pseudo R^2^ change (McFadden) = 0.02 (typically used in PROMIS analyses), as well as the Chi-square threshold (alpha = .01). In addition, we computed R^2^ thresholds suggested by Monte Carlo simulations (1000 replications, alpha =.01) under no DIF assumptions. We repeated lordif runs, at small increasing thresholds of R^2^ thresholds to examine which items consistently show the greatest overall DIF (uniform + non-uniform).

**Results**

No items were flagged for DIF with R^2^ change of 0.02. The Chi-square threshold, however, flagged all items for DIF. The maximum R^2^ value suggested by Monte Carlo simulations was 0.004; we re-ran the lordif function with incrementally higher R^2^ thresholds, starting with 0.005 and increasing to 0.015. Five of the 19 items showed consistently higher resulting values for R^2^ (> 0.01). These items showed mostly uniform DIF in the same direction, such that US participants were less likely to endorse items. The 4-item scale (PROMIS-29) contains two of these DIF items, while the 6-item scale (PROMIS-43) contains three. Language-specific location parameters were substantially different from one another (mean difference = .3 to .5).

**Conclusions**

We found preliminary evidence for consequential DIF. Small, uniform effect sizes (R2 ≈ 0.01) for two items can have a cumulative effect when the fixed-form is just 4-item long. Results demonstrate the value of applying Monte Carlo simulations to determine DIF thresholds. Limitations include the small German sample size.

**Keywords**

PROMIS, Physical Function, Language, Differential Item Functioning, Lordif, Simulation, Dutch, German, English

## P063 Are the Dutch-Flemish PROMIS Pain Interference and Pain Behavior Item Banks Essentially Unidimensional?

### Wouter Schuller^1,2^, Caroline B. Terwee^1^, Thomas Klausch^1^, Leo D. Roorda^3^, D.C. Rohrich^1^, Raymond W. Ostelo^1,4^, Berend Terluin^5^, Henrica C.W. de Vet^1^

#### ^1^ VU University Medical Center, Department of Epidemiology & Biostatistics and the Amsterdam Public Health Research Institute, P.O. box 7057, 1007 MB Amsterdam, The Netherlands; ^2^ Spine Clinic, Mahoniehout 10-12, 1507 ED, Zaandam, The Netherlands; ^3^ Amsterdam Rehabilitation Research Center, Reade, Doctor Jan van Breemenstraat 2, 1056 AB, Amsterdam, The Netherlands; ^4^ Department of Health Science of the Faculty of Earth and Life Sciences, VU University, De Boelelaan 1105, 1081 HV Amsterdam, The Netherlands; ^5^ Department of General Practice and Elderly Care Medicine, Amsterdam Public Health Research Institute, VU University Medical Center, van der Boechorststraat 7, 1081 BT Amsterdam, The Netherlands

**Background:** To study whether the Dutch-Flemish PROMIS Pain Interference and Pain Behavior item banks can be considered essentially unidimensional.

**Methods**

In a large sample of patients with musculoskeletal complaints we studied the dimensionality by confirmatory factor analyses (CFA) and by assessing local independency. A bi-factor model was used to identify sub factors, and Omega-H and Explained Common Variance (ECV) were calculated to assess whether multidimensionality was likely to lead to biased parameters. A Graded Response Model was used to study item fit, and to estimate slope and threshold parameters.

**Results**

The dimensionality of the Pain Interference item bank was evaluated in a sample of 1677 patients. We found evidence of suboptimal unidimensionality in CFA (CFI: 0.903, TLI: 0.897, RSMEA: 0.144), and 99 item pairs with local dependence. A bi-factor model showed good fit (CFI: 0.964, TLI: 0.961, RSMEA: 0.089), with a high Omega-H (0.97), a high ECV (0.81), and no local dependence. The GRM showed good fit for all but two items, slope parameters ranged from 1.00 to 4.27, and threshold parameters ranged from -1.77 to 3.66.

The dimensionality of the Pain behavior item bank was evaluated in a sample of 1602 patients. We found suboptimal evidence of unidimensionality in CFA (CFI: 0.816, TLI: 0.806, RSMEA: 0.093), and fifteen item pairs (2%) with local dependence. A bi-factor model showed better fit (CFI: 0.922, TLI: 0.915, RMSEA: 0.062), with a high Omega-H (0.92) and a high ECV (0.70). The GRM showed good item fit, slope parameters ranged from 0.60 to 2.00, and threshold parameters ranged from -2.05 to 6.80.

**Conclusions**

Despite evidence of suboptimal unidimensionality, the high Omega-H and ECV in bi-factor analyses indicated that the Pain Interference and Pain Behavior item banks can be considered essentially unidimensional.

**Keywords**

Pain Behavior item bank; Pain Interference item bank; bi-factor analysis; dimensionality;

## P064 A Study of Cognitive, Emotional, Physical and Socioeconomic Factors Associated with Patient-Reported Outcome Measures (PROMs) Completion Post-Stroke

### Alexander Smith¹, Jonathan Hewitt^1^, Terry Quinn^2^

#### ¹ Division of Population Medicine, Cardiff University, Cardiff, UK; ^2^ Institute of Cardiovascular and Medical Sciences, College of Medical, Veterinary & Life Sciences, University of Glasgow, Glasgow, UK

**Background**

The evidence base for Patient-Reported Outcomes use post-stroke either fails to report or sufficiently evidence appropriate levels of acceptability. Primary research into the factors that most affect PROM completion post-stroke is required to address insufficient PROM acceptability and to enable the reporting of perceptions of health, quality of life or the outcomes of treatment by stroke survivors.

**Methods**

Phase 1 will recruit participants following acute admission for cerebral infarction or intracerebral haemorrhage. Baseline Assessment (14 Days or less Post-Stroke) consists of a stroke-specific cognitive screen the Oxford Cognitive Screen (OCS) and an ultra-brief Anxiety and Depression Screen the PHQ-4. At 90 to 120 Days Post-Stroke, participants will be rescreened using the OCS & PHQ-4 before participants attempt to self-complete the PROMIS-10. In Phase 2 participants unable to fully complete the PROMIS-10 will be re-screened utilising the PHQ-4 & OCS and randomised 1:1 to self-complete either the original PROMIS-10 or an ‘Accessible’ PROMIS-10. The design of the ‘Accessible’ PROMIS-10 will be derived from an analysis of the acceptability of the original PROMIS-10 in responding to cognitive, emotional, physical and socioeconomic factors. The ‘Accessible’ PROMIS-10 will feature adaptations to aid completion such as; visual analogue scales (VAS), pictorial representations, larger font and bolding/ italicisation of key words. The primary outcome of the study is PROMIS-10 completion rate (number who attempt versus number that 100% complete). This will be analysed using a Chi-square test of independence of the Original PROMIS-10 completion rate against the Accessible PROMIS-10 completion rate (Alpha 0.05 and Power 95%). Secondary outcomes such as completion rate per question and as a percentage of questions completed will be analysed utilising Logistic or Linear regression.

**Results**

**Conclusions**

The study will provide evidence for which factors are related with the ability to complete PROMs post-stroke and will trial a method of addressing PROMs acceptability post-stroke.

**Keywords:** Stroke, CVA, Patient-reported Outcomes, PROMIS, Acceptability

## P065 Clinical Application and Usage of Patient Reported Outcome Measures in an Orthopaedic Outpatient Setting

### Amanda Spraggs-Hughes^1^, MA, Jason Guattery^2^, MS, Ryan P. Calfee^1^

#### ^1^Washington University in St. Louis, USA; ^2^University of Pittsburgh, USA

**Background**

The objective is to outline collection methods and clinical usage of PROMIS measures in the orthopaedic outpatient setting of an academic medical center.

**Methods**

The orthopaedic outpatient clinics of an academic medical center implemented the collection of PROMIS assessments in the summer of 2015. Orthopaedic faculty and midlevel providers were educated on the standard usage of PROMIS assessments and provided suggestions for adoption in clinical practice.

**Results**

Presently, PROMIS assessments are collected for the outpatient clinical visits of 60 orthopaedic faculty and 17 midlevel providers across 7 separate locations in a metropolitan area. PROMIS modules vary based on the population and the providers’ subspecialty. Assessments are delivered in computer adaptive testing (CAT) format and results are delivered in real time to the electronic medical record (EMR).This allows clinical providers to view a patient’s PROMIS score in the patient chart prior to the initiation of the patient visit. Pediatric PROMIS depression administration prompted parental complaints and resulted in our switch to delivery of pediatric peer relationships. Formal patient feedback regarding PROMIS scores has recently been delivered regarding adult Depression and Anxiety scores.

**Conclusions**

PROMIS has largely been incorporated into clinical practice successfully across practice locations. Depression CAT’s selectively caused more complaints for pediatric visits and that assessment was then discontinued. At this time, clinicians indicate that they may review Physical Function scores but no thresholds or rules are followed to allow those scores to change treatment. Given the inter-relationship between physical and mental health, our clinicians have now formulated formal handouts for patients to address heightened Anxiety and/or Depression scores at the time of clinical care. Such response to mental health scores was delayed from the start of survey use as processes required development and responses were established with additional input from the University’s Psychiatry Department and legal team.

**Keywords**

PROMIS, Patient Reported Outcomes, Orthopaedic Surgery

## P066 Research Applications of Patient Reported Outcome Measures in an Academic Orthopaedic Surgery Department

### Amanda Spraggs-Hughes^1^, MA, Jason Guattery^2^, MS, Ryan P. Calfee^1^

#### ^1^Washington University in St. Louis, USA; ^2^University of Pittsburgh, USA

**Background**

The primary aim is to describe the research output resulting from the implementation of routine PROMIS data collection in an academic orthopaedic surgery department.

**Methods**

The implementation of PRO data collection in the outpatient setting allowed for the collection of PROMIS assessments in computer adaptive testing (CAT) modules prior to patient visits. This informed the clinical decision making of the orthopaedic faculty but also introduced the capability to use that data to research orthopaedic conditions across subspecialties. Once approved by the local Institutional Review Board (IRB), our faculty were able to utilize PROMIS scores in research studies in specialized areas of focus.

**Results**

Presently, there are 5 peer reviewed journal articles in print resulting from our department’s PROMIS data collection. There are an additional 10 research manuscripts either in process or submitted pending review. At present, there are 31 IRB approved studies investigating PROMIS measures in a variety of musculoskeletal conditions. Interest in PROMIS data collection and reporting also prompted the formation of a PROMIS study group within the department. This group’s purpose is to share best practices in PRO related research methodology and statistical analysis.

**Conclusions**

The introduction of PROMIS data collection in the outpatient clinics at our institution increased the usage and reporting of research related findings in musculoskeletal injuries and conditions. Aided by an enthusiastic department chairman and widespread institutional support, our clinical faculty’s interest in collecting and reporting PROMIS data has increased precipitously. The large demand for PRO data within research studies has also spurred further development of WUPRO to include greater research functionality. This functionality includes multiple methods of REDCap integration including direct data transfer to REDCap studies and integrating research forms alongside clinical data which will further increase our ability to leverage clinical PRO data collection to support research applications.

**Keywords**

PROMIS, Patient Reported Outcomes, Orthopaedic Surgery

## P067 Using Individualized PROMIS^®^ Short Forms to Measure the Effect Of Personalized E-Health Perioperative Care on Postoperative Recovery After Abdominal Surgery

### Eva van der Meij^1,3^, Johannes R Anema^1^, Caroline B Terwee^2^, Judith AF Huirne^1,3^ (on behalf of the study team)

#### ^1^Department of Public and Occupational Health; ^2^Department of Obstetrics and Gynaecology; ^3^Department of Epidemiology and Biostatistics, VU University Medical Center, Amsterdam, The Netherlands

**Background**

We aim to show how PROMIS item banks can be used to develop individualized short forms to measure more relevant outcomes for patients.

**Methods**

The effect of a new e-health intervention on return to normal activities was evaluated by a randomized controlled trial in 344 patients undergoing various types of abdominal surgery. Because “normal activities” is different for each patient, an individualized outcome measure was used. Participants were asked to select at baseline eight items from a pre-selected list of 29 items from the PROMIS V1.2 Physical Function item bank, which in their view most reflected their day-to-day activities. A T-score on the PROMIS metric was calculated for each patient based on their selected eight items. At follow-up patients completed the same eight items and indicated whether or not they had resumed these activities and if so, since when. The moment on which the last activity was resumed was the primary outcome of the trial. The T-score at follow-up (corrected for baseline) was the secondary outcome measure.

**Results**

Median time until return to normal activities was 5 days shorter for participants in the intervention group as compared to the control group (p=0.011). The mean physical function T-score was significantly higher at follow-up in the intervention group compared with the control group (0.024).

**Conclusions**

This is the first study that evaluated the effect of e-health on return to normal activities after abdominal surgery. Unique was the use of an individualized outcome measure, taking advantage of validated IRT-based item banks. Participants selected those activities that were most relevant for them in daily life and thus return to normal activities was specific to the outcomes that matter to participants. In addition, the selected activities matched the personalized e-health intervention. As a consequence, the effect of the intervention could be measured very specifically.

**Keywords**

Randomized Controlled Trial, E-Health, Patient-Reported Outcomes, IRT, PROMIS

## O068 Is PROMIS^®^ Promising in Rehabilitation? A Pilot Study in Regular Care

### F.M. van Vree ^1,2^, G. Volker ^1^, A.J. de Kloet ^2,4^, I.F. Groeneveld ^1,2^, J.J.L. Meesters ^2,3^,T.P.M. Vliet Vlieland ^1,2,3^, P.H. Goossens ^1,3^

#### ^1^ Rijnlands Rehabilitation Center, Leiden, the Netherlands; ^2^ Sophia Rehabilitation Center, the Hague, the Netherlands; ^3^ Leiden University Medical Center, Leiden, the Netherlands; ^4^ The Hague University of Applied Sciences, the Hague, the Netherlands

**Objective**

To examine the feasibility of the Patient-Reported Outcomes Measurement Information System (PROMIS) in patients with various diagnoses in an outpatient rehabilitation setting.

**Methods**

Single centre pilot study, using PROMIS short forms collected online, including the PROMIS Ability to Participate in Social roles and activities (PROMIS-APS) and PROMIS Satisfaction with Participation in Social roles (PROMIS-SPS). These scales have a range from 0 to 100, where 50 depicts the average of the US population. Data were obtained at start of outpatient rehabilitation between April 12th 2017 and August 20th 2018 in the Rijnlands Rehabilitation Centre in Leiden, The Netherlands. Patients’ age, diagnosis and sex were derived from electronic patient registries. Mean PROMIS-APS and SPS t-scores and SD were calculated and compared between diagnosis, age groups and gender using T-tests, One-Way Anova and multivariable regression analysis (GLM).

**Results**

639 patients completed the electronic versions of the PROMIS-APS and SPS scales (no reminders sent, +/-50% response): 304 with acquired brain injury (Mean PROMIS-APS; PROMIS-SPS 43,0;41,6), 92 with neuro muscular diseases (43,4;43,0), 88 with spinal and nerve injury (42,6;41,8), 61 with musculoskeletal disease (42,1;41,2), 40 with organ rehabilitation (43,9;41,4), 29 with chronic pain (40,0;38,8) and 22 with amputation (43,2;39,3). Mean (±SD) age of participants was 55 years (±15), 321 (50%) were male. Mean (+SD) PROMIS-APS t-score was 42,8 (+7,5) and PROMIS-SPS 41,6 (+7,7). Sex was significantly associated with PROMIS-APS and with PROMIS-SPS t-scores. Differences in means between diagnosis were not significant.

**Conclusion**

The PROMIS-APS and PROMIS-SPS scales were successfully completed online by half of the patients admitted for outpatient rehabilitation, without sending reminders. The PROMIS-APS and PROMIS-SPS t-scores were significantly lower than those of the US general population within all diagnosis groups. Preliminary data showing changes overtime will be presented.

## P069 Collection of PROMIS^®^ Measures Using a Digital Platform. Interim Results From a Feasibility Study

### T. Waters^1^, M. Ries^2^, G. Datta^3^, E. Davis^4^, D. Nathwani^5^, P. Sutton^6^, A. Bernstein^7^, A. Turco^8^, D. De Faoite^8^, I. McNamara^9^

#### ^1^St. Albans City Hospital, Waverley Rd, St Albans AL3 5PN, UK; ^2^Reno Orthopedic Clinic, 555 N Arlington Ave, Reno, NV 89503, US; ^3^University Hospital Southampton, Tremona Rd, Southampton SO16 6YD, UK; ^4^The Dudley Group NHS Foundation Trust, Russels Hall, Pensnett Rd, Dudley DY1 2HQ, UK; ^5^Imperial College London, Charing Cross Campus, Margravine Rd, Hammersmith, London W6 8RP, UK; ^6^Sheffield Teaching Hospitals NHS Foundation Trust, Glossop Rd, Sheffield S10 2JF, UK; ^7^Wellframe Inc., 321 Summer Street, Boston, MA 02210; ^8^Smith & Nephew, Global Clinical Strategy, Oberneuhofstrasse 10d, 6340 Baar, Switzerland; ^9^Norfolk and Norwich University Hospital, Colney Lane, Norwich, NR4 7UY, UK

**Background**

Acquisition, interpretation and dissemination of clinical data are driving change throughout the global healthcare market. Introduction of new regulatory requirements also places increased emphasis on the need to generate relevant clinical data. A digital platform to enhance patient engagement and collect patient-reported outcome measurements (PROMs) was recently adapted for TKA patients. A 4-month long feasibility study was initiated to gather feedback on the usability of this digital platform.

**Methods**

The digital platform consists of a patient mobile application (app) and a clinician dashboard. The app collects a variety of PROMs, including some based on the Patient-Reported Outcome Information System (PROMIS®). Additional features of the digital platform include staff-patient messaging, reminders and educational articles.

Fifty-two patients (mean age 62.8 years, 56% females) were enrolled from 5 UK sites and 1 US site from January 12, 2018 to April 19, 2018. This work reports upon the results from this initial cohort of patients in terms of patient engagement and PROMIS® CAT surveys completion. Four PROMIS® domains were measured: Physical Function, Depression, Pain Interference and Pain Behaviour. Depending on their post-operative phase, a subset of patients additionally responded to surveys to assess app user experience.

**Results**

The patients demonstrated willingness to engage with the platform. On average, 83% of all enrolled patients engaged with the app at least once per week. Patients completed 77% of all PROMIS® CAT surveys during the considered timeframe, with similar survey completion rates regardless of the scoped PROMIS® CAT domain. Patients that completed the user experience questions responded a mean of 8.9/10 for the ease-of-use of the app (n=22). 9/13 patients were successful (7/10 or higher) at using the information from the app during their recovery.

**Conclusions**

The results so far collected show high patient engagement, app satisfaction and high adherence to the PROMIS® CAT survey completion.

**Keywords**

Patient Engagement, PROMIS® CAT, Physical Function, Depression, Pain Interference, Pain Behaviour, Clinical Research.

## P070 PROMIS^®^ Emotional Distress Scores as Cues for Action

### Lari Wenzel, Jo Anne Tucker, Chelsea O McKinney, Dara Sorkin, Kathryn Osann, Edward L Nelson

#### University of California, Irvine, USA

**Objective**

To determine if oncologists would independently utilize PROMIS scores as cues to action when patients’ scores signaled emotional distress.

**Methods**

We used the static PROMIS-Emotional Distress fixed-length paper-pencil self-administered short forms for depression (8 items) and anxiety (8 items) during consecutive outpatient oncology visits from 2013 through 2017. Forms were scored by the medical assistant during the patient visit, and provided to the clinician for review and follow-up, as indicated. Clinicians were notified that a raw score equivalent to a T-score > 55 (i.e. at least 0.5 SD higher than the mean) warrant attention.

**Results**

PROMIS forms were completed during 12,526 patient encounters, yielding 4,569 unique patients with at least one completed PROMIS assessment. Among these unique patients, 26% reported mild to severe distress. Among those who exhibited distress, 19% received an action of any type, as noted in the chart. Women who experienced mild to severe distress were significantly more likely to receive an action at 24% compared to their male counterparts at 12% (*p* < 0.000). Patients of color, specifically Hispanic and Asian patients, were more likely to report mild to severe distress (OR: 2.08 and 1.78 respectively, *p* < 0.000) even after adjusting for gender and age. Hispanic and Asian patients were also more than twice as likely to receive an action from providers given their scores (OR: 2.58 and 2.12 respectively, *p* < 0.000). Of the total providers, 33% administered actions when presented with informative distress trigger scores.

**Conclusions**

PROMIS emotional distress scores can provide meaningful prompts to action that address mental health concerns in the general oncology outpatient setting. Findings also highlight clinicians’ capacity to address a racial/ethnic disparity in reporting distress among cancer patients. However, coaching, system-wide endorsement and electronic health record score integration is needed for these scores to be actively utilized.

**Keywords**

Emotional Distress, Depression, Anxiety, Oncology, Cancer, Outpatient, Action, Disparity

## P071 Social Deprivation's Impact on Physical and Mental Health in Orthopedic Patients

### Melissa Wright, Casey M. Beleckas, BS; Muyibat Adelani, Christopher Dy, MPH, Regis O'Keefe, Ryan P. Calfee

#### Washington University School of Medicine , USA

**Background**

This study assessed the impact of social deprivation on PROMIS Physical Function, Pain Interference Depression, and Anxiety scores in patients presenting for orthopedic care.

**Methods**

This cross sectional evaluation analyzed 7,500 new adult patients presenting to an orthopedic center between August 1, 2016 and December 15, 2016. Patients completed PROMIS Physical Function-v1.2, Pain Interference-v1.1, Depression-v1.0, and Anxiety-v1.0 Computer Adaptive Tests. The area deprivation index quantified social deprivation. Statistical analysis determined the effect of disparate area deprivation (based on most and least deprived national quartiles) for the entire population as well as patients categorized by the orthopaedic subspecialty providing care.

**Results**

Patients living in the most deprived quartile had significantly worse mean scores across all four PROMIS domains when compared to those living in the least deprived quartile, (p<0.01). Significant differences on PROMIS domains according to deprivation quartile was not evident for patients cared for by the Trauma, Oncology, and Spine divisions, where PROMIS scores indicated poorer physical and mental health than patients seeing other specialists.

**Conclusions**

Patients from areas of high social deprivation have worse PROMIS Physical Function, Pain Interference, Depression, and Anxiety scores at presentation for orthopedic care. However, in select patient populations with the worst baseline mental and physical health scores, social deprivation does not further impact patient-reported health.

**Keywords**

PROMIS, Orthopedic, Social Deprivation, Socioeconomic

## O072 Attitudes, Experiences, and Willingness to Use Of PRO Measures Among Health Professionals In Oncology

### Junghee Yoon^1,2^, Youngha Kim, MS^1^, Dongryul Oh ^3^, Mangyeong Lee, MS ^2^, Seonhwa Park^1^, Juhee Cho^1^

#### ^1^ Center for Clinical Epidemiology, Samsung Medical Center, Seoul, Korea; ^2^ Department of Digital Health, SAIHST, Sungkyunkwan Univesrity, Seoul, Korea; ^3^ Department of Radiation Oncology, Samsung Medical Center, Seoul, Korea

**Background**

Patient-reported outcome (PRO) has become an emerging outcome in oncology practice. However, a little is known about health professionals’ attitudes toward PRO and using experience. We aim to evaluate attitudes, experiences, and willingness to use of PRO among physicians and nurses work with cancer patients in Korea.

**Methods**

This is a cross-sectional survey conducted with 139 physicians and 71 nurses from August to September 2017 in Korea. Health professionals were recruited at the major cancer conference in Korea. Ten questions were asked to assess attitudes, knowledge, experience, and willingness to use the PRO for research and patient care. We also evaluated health professionals’ needs and willingness to participate in education and training to learn about the assessment of PRO.

**Results**

Of total, 27.6% of the study participants said that they were exposed to the PRO and most of them learn about it from academic meetings or publication. Health professionals consider themselves that they do not have appropriate knowledge about the PRO (2.9 out of 10, 0=no knowledge, 10= high knowledge) and majority participants (73.8%) had intention to use PRO both for clinical care and research. In multivariate analysis, being nurse, specializing medical oncology, and working at academic institution were positively associated with experience with PRO. Most of the study participants (88.1%) have willingness to have education about PRO.

**Conclusions**

While oncology health professionals had limited experience, they had positive attitudes towards the PRO suggesting the needs of education and training for the health professionals.

**Keywords**

Patient-Reported Outcomes, attitudes, oncology, education

## O073 Development and Validation of the PROMIS^®^-Plus OA-K Profile Measure

### Susan E. Yount^1^, Michael A. Kallen^1^, Karen E. Schifferdecker^2^, Kathleen L. Carluzzo^2^, Lynn M. Marshall^3^, Kathryn Schabel^3^, William Robb^4^, David W. Manning^1^, Elliott S. Fisher^2^, David Cella^1^

#### ^1^Feinberg School of Medicine, Northwestern University, Chicago, IL, USA; ^2^Geisel School of Medicine, Dartmouth College, Lebanon, NH, USA; ^3^Oregon Health and Science University, Portland, OR, USA; ^4^Illinois Bone and Joint Institute, Glenview, IL; Pritzker School of Medicine, University of Chicago, Chicago, IL, USA

**Background**

We sought to tailor a set of universal patient-reported outcomes measures (The Patient-Reported Outcomes Measurement Information System^®^; PROMIS^®^) by integrating items specific to osteoarthritis of the knee (OA-K), thus facilitating comparisons to other conditions and the general population while enhancing OA-K sensitivity and clinical relevance.

**Methods**

Eight focus groups with OA-K patients (N=68) and phone interviews with clinicians (N=6) were conducted. Through an iterative process, existing items were reviewed, new items drafted, and items revised and cognitively tested (N=10 patients). In a cross-sectional sample of OA-K patients, we estimated reliability (internal consistency N=600; test-retest subsample N=100). We conducted convergent/divergent validity analyses using Pearson *r* and Spearman *rho* correlations with Knee Injury and Osteoarthritis Outcome Score (KOOS) subscores and known-groups validity testing with PROMIS Global Health (high vs. low Physical and Mental status). Measure responsiveness was tested via paired t-tests in a longitudinal sample of 238 OA-K patients pre-/post-total knee replacement.

**Results**

PROMIS-Plus OA-K includes 76 items from 14 domains: 52 existing PROMIS items and 24 new OA-K-specific items. For cross-sectional analyses, internal consistency reliability (Cronbach’s alpha) was 0.67-0.95, with alpha ≥0.70 in 10 of 12 domains. Test-retest reliability (intraclass correlation coefficients) were all ≥0.90. Correlations with KOOS subscores and PROMIS Global supported expected convergent (*r*/*rho* >0.60) and divergent validity (*r*/*rho* <0.30). We demonstrated known-groups validity with evidence of better health status in all PROMIS-Plus OA-K domains for high Global Physical and Mental status groups compared to low status groups. In our longitudinal sample, all PROMIS-Plus OA-K domains had statistically significantly (*p*<0.001) better health status scores at follow up vs. baseline.

**Conclusions**

The PROMIS-Plus OA-K Profile demonstrated good psychometric characteristics. The measure’s enhanced relevance to OA-K patients may facilitate patient-centered care and research. Select domains can be administered, based on the preference and aims of the clinician, researcher and patient.

**Keywords**

Patient-Reported Outcomes, Knee Osteoarthritis, PROMIS

## O074 Validation of PROMIS^®^ Profile-29 v2.0 (Polish version) Among Orthopedic Patients

### Żukowska A.^1,2,4^, Dymitrowicz M.^1,2,4^, Glinkowski W.^1,2,3,4,5^

#### ^1^ Polish Telemedicine and eHealth Society, Warsaw, Poland; ^2^ Center of Excellence “TeleOrto”, for Telediagnostics and Treatment of Injuries and Disorders of the Locomotor System, Medical University of Warsaw, Poland, 3 Gabinet Lekarski, Warsaw, Poland; ^4^ polskipromis.pl; ^5^Faculty of Health Sciences and Physical Education, Kazimierz Pulaski University of Technology and Humanities in Radom, Radom, Poland

**Objective**

Many patient-reported outcome (PRO) instruments used in musculoskeletal disorders and injuries trials in Poland are limited by lack of validation, licensing fees, and complicated scoring systems. We assessed the construct validity for discriminative purposes of the Patient-Reported Outcomes Measurement Information System 29-item Health Profile (PROMIS-29), measuring health status in orthopedic patients. Patient Reported Outcomes Measurement Information System (PROMIS®) provides standardized measures across domains of physical, mental, and social health. Patients with orthopedic diseases and dysfunctions suffer not only form physical limitations and pain but also from depression. This study evaluated the reliability and construct validity of PROMIS Profile-29 v.2.0 (Polish version) instrument.

**Patients and Methods**

Patients suffering from the degenerative joint disease (osteoarthritis of the knee, hip, and spine) were enrolled in the study. The translation from the English language to the Polish language, synthesis, back-translation, revision and cognitive testing were performed. Paper-and-pencil version of PROMIS Profile-29 v.2.0 (Polish version) was used. The test-retest the questionnaire was administered twice with 24-72 hours time interval. Data were analyzed with Scoring Service provided by Assessment Center (www.assessmentcenter.net), and PROMIS Wave 1 was chosen as a calibration sample. Each domain of PROMIS Profile-29 is scored individually. Physical Function, Pain Interference, and Depression domains were selected as the most relevant for orthopedic patients with the degenerative joint disease. Internal consistency of the translated questionnaire was tested by Cronbach's alpha, test-retest reliability was examined by the intra-class correlation coefficient (ICC).

**Results**

Ninety patients with the degenerative joint disease (osteoarthritis of the knee, hip, and spine) participated in a cross-sectional study at orthopedic and orthopedic rehabilitation outpatient clinics completed the Polish version of the PROMIS Profile-29 v.2.0. The group consisted of 39 men and 51 women. The average age was 66 years (34 ÷ 85 yrs; ± 12 yrs ). Women average age was 65 years (40 ÷ 83 yrs; ± 12 yrs ), men average age was 68 years (34 ÷ 85 yrs; ± 12 yrs ). Internal consistency tested by the Cronbach's alpha (0.82 – 0.93) was good to excellent. The intra-class correlation coefficient was R = 0,90 (95% confidence interval: 0.04 – 0,11).

**Conclusion**

The results showed that PROMIS Profile-29 v.2.0 (Polish version) is a valid tool to utilize among orthopedic patients with the degenerative joint disease. The measurement instrument has good to excellent reported test-retest reliability measured over durations of time ranging from 1 to 3 days. PROMIS Profile-29 v.2.0 (Polish version) is an effective tool to measure patient-reported outcomes and feasible to use in clinical trials in Poland.

**Keywords**

Orthopedic Patients, Musculoskeletal Disorsders, Degenerative Joint Disease, Osteoarthritis, PROMIS Profile-29 V.2.0 - Polish Version

